# Quantification of small (1–10 µm) microplastic particles in soil matrices using automated scanning electron microscopy: possibilities and limitations

**DOI:** 10.1007/s00216-025-06111-8

**Published:** 2025-10-14

**Authors:** Ralf Kaegi, Matthias Philipp, Isabel S. Jüngling, Natalia P. Ivleva, Thomas D. Bucheli

**Affiliations:** 1 Swiss Federal Institute of Aquatic Science and Technology, Überlandstrasse 133, CH-8600 Dübendorf, Switzerland; 2 Chair of Analytical Chemistry and Water Chemistry, Institute of Water Chemistry (IWC), TUM School of Natural Sciences (NAT, Dep. Chemistry), Technical University of Munich, Lichtenbergstraße 4, 85748 Garching, Germany; 3 Agroscope Environmental Analytics, 8046 Zurich, Switzerland

**Keywords:** Single particle analysis, Automated electron microscopy, Microplastics, Monte Carlo simulation, Sample preparation

## Abstract

Scanning electron microscopy (SEM) provides superb resolution power and allows distinguishing microplastic particles (MPs) from other particles based on elemental ratios derived from energy dispersive x-ray (EDX) analysis. Polycarbonate (PC) membranes are typically used to isolate MPs from suspensions for SEM investigations. This, however, results in poor (atomic weight) contrast between MPs and the underlying PC membrane, challenging automated particle detection. To introduce an elemental contrast between MPs and the PC membrane, a gold (Au) coating was applied to the PC membrane before use. Monte Carlo simulations conducted to optimize operational conditions of the SEM suggest an acceleration voltage of 3 kV in combination with a 40 nm Au coating of PC membranes as most appropriate. Stock suspensions made from polyethylene (PE) fragments, polyvinyl chloride (PVC) fragments, and standard soils, all sieved to 0.45–10 µm, were mixed at different ratios. Selected mixtures were density separated to isolate the MPs from the mixtures. Whereas up to 7% of PE was detected in PVC stocks, likely representing false positives, only negligible amounts of PVC were detected in PE stocks. In soils, neither PE nor PVC was detected in significant amounts. Polyethylene and PVC particles diluted in soil suspensions at particle number ratios of 1:1:2 and 1:1:2000 were density separated, and largely concentration independent recoveries of around 70% and 50% were obtained for PE and PVC, respectively. Good agreement between SEM-EDX and Raman measurements was obtained for MPs, underlining the strength of our approach to address the smallest size fraction of MPs in complex matrices.

**Graphical abstract:**

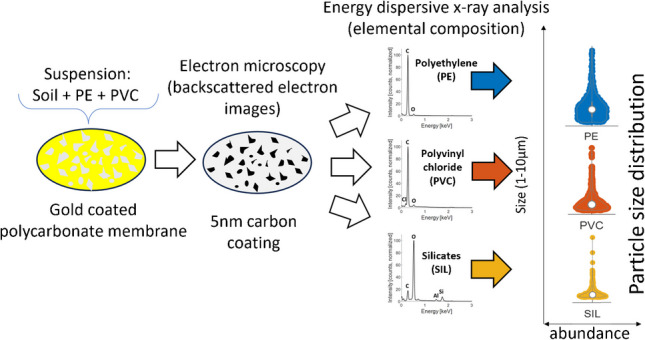

**Supplementary Information:**

The online version contains supplementary material available at 10.1007/s00216-025-06111-8.

## Introduction

Since the onset of mass production of plastics in the 1950 s, the annual production volume of these materials has exceeded 400 mio metric tons already in 2015 [1]. Their manifold and modifiable properties are key to their successful applications in diverse fields ranging from transportation over construction to healthcare [2]. However, concerns about the unsustainable use of polymers in general have been raised more than a decade ago [3]. As of 2015, estimations suggest that up to 60% of all plastics produced were discarded and are now accumulating either in landfills or in the open environment [1]. Environmental weathering, most prominently photochemical and mechanical aging, leads to an embrittlement of the polymers through molecular chain scission, and thus to a fragmentation [4–8], resulting in the production of smaller plastic fragments (microplastic particles (MPs) when < 5 mm and nanoplastic when < 1 µm). As a consequence, MPs have been reported from all over the globe including distant locations such as Antarctica, the deep ocean, and remote mountainous areas [9–14].

The concentration of microplastic in a given matrix is either reported as the mass of individual polymers (mass-based) or as the number and size of individual MPs (particle-based) detected in the respective matrices [15–19]. Due to the increasing likelihood for uptake of MPs by organisms with decreasing particle size, particle-based methods especially addressing the smallest size fraction down to 1 µm are urgently needed [20]. However, available methods that can cover MPs down to 1 µm are confronted with several challenges and are, thus, only poorly established, yet, as outlined in the following paragraph.

Currently used particle-based methods mostly rely on vibrational spectroscopy techniques such as µ-Fourier transform infrared (FTIR) and Raman spectroscopy [18, 21, 22]. Both techniques require the isolation of the particle of interest and the deposition on a suitable substrate. Due to diffraction limitations, the detection limit for FTIR-based instrumentation is around 10–20 µm [18] and the method is therefore not suitable for assessing the smallest (sub 10 µm) size range of MPs. As the Raman technology uses considerably shorter (laser) wavelengths, the theoretical detection limit is slightly below 1 µm [22]. Raman imaging refers to a point-by-point analysis and is thus very time-consuming [23]. To cover an area of 0.8 × 0.8µm^2^ at a spatial resolution of 1.1 µm, ~ 12 h of measurement time was required [24]. A particle-by-particle analysis is less time-intensive, but essentially relies on accurate optical detection of the particles [25–27], which can be challenging when dealing with differently colored or translucent particles. Improvements in particle detection have recently been achieved by implementing several image analysis tools into the analysis workflow [28, 29]. Chemical treatments and the choice of suitable sample substrates can be applied to mitigate fluorescent effects and improve automated particle detection [22, 30]. However, the more demanding the sample treatment, the more prone it is to particle losses.

In addition to the Raman spectroscopy, the use of fluorescent tags to selectively stain MPs offers the possibility to detect MPs in the lower micron size range [31–34]. This approach, however, was criticized due to insufficient specificity [35]. Furthermore, the interaction of the staining agent (Nile red) with organic matter and polymer-type specific staining efficiency challenged the quantification of stained MPs using flow cytometry [36]. Promising results for the detection of MPs around 1 µm were recently presented using optical photothermal infrared spectroscopy [37–40], however, with still a limited degree of automation. Other emerging techniques such as stimulated Raman [41–43] recently also combined with flow cytometry [44], surface-enhanced Raman spectroscopy [45], and MP detection through a combination of optical or electrical tweezers with Raman spectroscopy [46–49] also hold great promise in assessing MPs in the sub 10 µm size range. These methods, however, are still in their early development phase, and published results largely reflect feasibility studies conducted on model particles in clean matrices.

The lack of established analytical methods with documented particle-based recoveries for MPs in the sub 10 µm size, therefore, calls for alternative analytical approaches to assess the lower particle size range of MPs (1–10 µm). Electron microscopy provides superior spatial resolution and has therefore frequently been used to characterize the surface morphology of isolated MPs in detail and/or to confirm their polymeric nature based on energy-dispersive x-ray (EDX) analysis [e.g., 50–52]. Furthermore, electron microscopy techniques also provide largely automated routines for particle analysis. First reports on computer-controlled or automated scanning electron microscopes (SEM) for particle analysis date back to 1969 [53]. Since then, the capabilities of automated SEM have continuously been expanded, and EDX analysis of individual particles has been included in the respective hardware environments and software tools. Semi-automated algorithms were developed specifically addressing atmospheric particles composed of light elements such as nitrates, carbonates, and sulfates, using an electron probe microanalyzer equipped with an EDX system [54–58]. Furthermore, the aging of individual particles, especially sulfates, nitrates, carbonates, carbonaceous particles, and chlorides, during their transport in the atmosphere was intensively investigated by automated SEM in combination with elemental analysis (EDX) [59–63]. Complementary to these mostly volatile (i.e., degrading/volatilizing under the electron beam) particles, refractory aerosol particles were also studied in great detail using automated and operator-controlled SEM-EDX approaches [e.g., 62–68]. Most recently, Li et al. [69] reviewed the possibilities of automated SEM in atmospheric particle research and suggested focusing on improving the detection of particles with low atomic numbers.

To limit the contribution of backscattered electrons and x-rays from the substrate, carbon (C)-coated transmission electron microscope (TEM) grids (e.g., [59, 60, 70]) as well as polished boron (B) crystals (e.g., [57, 65]) were successfully used as sample substrates. Unfortunately, such substrates are incompatible with filtration approaches, which are required to isolate and representatively deposit MPs from suspension on respective substrates. Polycarbonate (PC) membranes provide flat surfaces with well-defined pore sizes and are therefore commonly used to isolate MPs from suspensions for SEM investigations. However, the use of PC membranes as substrates results in a poor (atomic weight) contrast against MPs and leads to the emission of C x-rays, both challenging the (automated) MP detection and classification. Coating the PC membranes with a thin layer of gold (Au) prior to its use still allows the isolation of MPs by filtration, eliminates the carbon background and at the same time, and provides a strong (elemental) contrast between the MPs and the underlying Au coating, facilitating the automated detection of the MPs. Such a novel setup, however, requires a reassessment of optimal operational conditions of the SEM to match the needs for MP analysis in soil matrices.

We, therefore, first derived optimal operational conditions for detecting and analyzing MPs in the size range between 1 and 10 µm filtered on Au-coated PC membranes based on Monte Carlo simulations and corresponding analyses. We then determined the dynamic range of the method using polyethylene (PE), polyvinyl chloride (PVC), and soil suspensions mixed at different ratios. As MPs are expected to occur at trace concentrations in soils, we further implemented a density separation protocol to isolate (diluted) MPs from spiked soil matrices to illustrate the potential of our approach in quantifying MPs in complex environmental samples. Additional Raman microspectroscopy measurements were conducted on duplicate samples to validate our analytical approach.

## Materials and methods

### Chemicals and devices

All samples were prepared under a laminar flow bench. The rinsing solution (0.02% sodium dodecyl sulfate (SDS) in ultrahigh purity water (Arius Pro, Sartorius, Göttingen, Germany)) and the sodium polytungstate (SPT) solution (1.6 g/mL, sodium metatungstate hydrate, crystalline; VWR International GmbH, Dietikon, Switzerland) were filtered through PC membranes (47 mm diameter, 0.05 µm pore size, Whatman, OH, USA) before use. Ethanol (EtOH, purity > 99.8%, Reuss Chemie, Tägerig, Switzerland) and isopropanol (IPA, purity > 99.8%, VWR) were used as received. All vessels and glass vacuum filtration units (VWR) used were cleaned with acetone (technical quality, Reuss Chemie) and dried with compressed air before use. For C coating and Au sputtering, a CCU-010 carbon coater (safematic, Zizers, Switzerland) and a EM ACE600 sputter coater (Leica Microsystems GmbH, Wetzlar, Germany) were used. For size assessments, 1-µm polystyrene (PS) beads (Polybead® Microspheres (No: 07310-15), Polysciences, USA) were used.

### Preparation of the stock suspensions

Polyethylene and PVC particles were received from Nanofrac (https://www.nanofract.com/, Germany) in suspension. Results from attenuated total reflection (ATR) FTIR measurements were consistent with the structure of PVC and PE, respectively, and additional µ x-ray fluorescence (XRF) measurements revealed small amounts of calcium in the PE pellets, which were ground to produce the PE particle suspensions (SI section S1, Fig. S1 and S2, Table S1). This will be further discussed in the “Particle identification and classification” section. LUFA standard soils (LUFA, Speyer, Germany) and quartz sand (< 0.6 mm, Krone-Gips Quarzsand, Germany) were ground in a ball mill using stainless steel buckets (MM400, Retsch, Haan, Germany). All particulate materials were thereafter suspended in organic solvents (PE, soil, and sand in EtOH; PVC in IPA). These suspensions were sequentially filtered through a stainless-steel mesh (MINIMESH® RPD HIFLO-S, 47 mm diameter, 10 µm pore size, Haver Boecker, Oelde, Germany) and a PC membrane (47 mm diameter, 0.45 µm pore size) to obtain particle fractions between 0.45 and 10 µm. After filtration, the particles were rinsed from the PC membranes into either the organic solvents (stock series 1 and 2; PE, soil, and sand in EtOH, PVC in IPA) or into the SPT solution (stock series 3) using the rinsing solution. All stock suspensions were stored in Schott glass bottles at room temperature in the laboratory.

Preliminary particle number concentrations of the stock suspensions were determined using a light microscope (VHX7000, Keyence, Japan). For this purpose, defined volumes of the individual stock suspensions were filtered on PC membranes (25 mm diameter, 0.2 µm pore size) and imaged using the light microscope (0.04% of each filter imaged at 2000× magnification). The particles were identified, and their number quantified using image analysis tools (software bundle associated with VHX7000, Keyence). These rough estimates from light microscopy analysis were used as a guide to determine the volumes of the stock suspensions needed to prepare the mixtures for subsequent experiments. Definitive particle number concentrations of the stock suspensions as well as of all experimental samples were subsequently derived from automated SEM measurements.

### Preparing experimental suspensions for automated SEM analysis

The composition of the experimental suspensions is listed in Table 1, and all sample numbers in the manuscript refer to this table. All experimental suspensions were filtered on PC membranes (25 mm diameter, 0.10 µm pore size) which were coated with a 40-nm Au layer prior to use. Before analysis in the SEM, the loaded PC membranes were coated with 5 nm of C. Recoveries of close to 90% were obtained from experiments using PS suspensions with a certified particle number concentration (Count Check Beads, No: 05-4011_R, Sysmex Partec GmbH). Given an uncertainty of ± 10% reported for the number concentration in the certified PS suspension, MP losses associated with filtration and filter handling (real losses) and apparent losses related to the particle detection in the electron microscope were considered negligible (SI, Section S2, Table S2).

**Table 1:** Composition of the experimental samples. Samples 1–3 and 7–8, prepared from stock series 1, were used to develop the classification scheme and to assess the dynamic range of the method. Samples 4–6 represent additional soil samples used to assess possible false positives in soil extracts. Sample 9, prepared from stock series 2, was used for a comparison between automated scanning electron microscopy and automated Raman microspectroscopy measurements. Samples 10–14, prepared from stock series 3, were used to assess the recoveries of the method. “extracted” refers to samples, where microplastic particles were extracted from suspensions using a density separation process. In total, 8 mL of either ethanol (EtOH) or sodium polytungstate (SPT) was used to dilute all experimental suspensions. PE, polyethylene; PVC, polyvinyl chloride; IPA, isopropanol

Number	Description	Stock (PE) (µL)	Stock (PVC) (µL)	Stock (SOIL) (µL)	Nominal particle number ratio (PE:PVC:SOIL)^f^	Liquid	Density sep
1^a^	PE pure	200	0	0	-	EtOH	NO
2^a^	PVC pure	0	50	0	-	IPA	NO
3^a^	SOIL (Lufa 2.2)	0	0	10	-	EtOH	NO
4^a^	SOIL (Lufa 2.4)	0	0	100	-	EtOH	NO
5^a^	SOIL (Lufa 5 M)	0	0	20	-	EtOH	NO
6^a^	SOIL (Lufa 6S)	0	0	100	-	EtOH	NO
7^a^	PE-PVC-SOIL (1:1:2)	200	50	20	1:1:2	EtOH	NO
8^a^	PE-PVC-SOIL (1:1:20)	200	50	200	1:1:20	EtOH	NO
9^b^	PE-PVC-SAND (2:1:3)^d^	750	150	20,000	2:1:3	EtOH	NO
10^c^	PE-PVC-SOIL in SPT (1:1:2)^e^	300	250	2.6	1:1:2	SPT	NO
11^c^	PE-PVC-SOIL in SPT (1:1:2)^e^	300	250	2.6	1:1:2	SPT	NO
12^c^	PE-PVC-SOIL in SPT(1:1:2)^e^	300	250	2.6	1:1:2	SPT	NO
13^c^	PE-PVC-SOIL in SPT (1:1:2), extracted	300	250	2.6	1:1:2	SPT	YES
14^c^	PE-PVC-SOIL in SPT (1:1:2000), extracted	300	250	2600	1:1:2000	SPT	YES

^a^Stock series 1: Individual suspensions of PE (~ 3000#/µL), PVC (~ 9000 #/µL), and soil particles (~ 60,000 #/µL); PE and soil prepared in EtOH, PVC prepared in IPA

^b^Stock series 2: Individual suspensions of PE (~ 500#/µL), PVC (~ 1400 #/µL), and sand particles (~ 14 #/µL); PE and sand prepared in EtOH, PVC prepared in isopropanol IPA. Stocks made with sieved sand instead of soils and used for comparison with Raman measurements

^c^Stock series 3: Individual suspensions of PE (~ 800#/µL), PVC (~ 1,000 #/µL), and soil particles (~ 200,000 #/µL); all prepared in SPT solution (density 1.6 g/mL)

^d^Due to a shortage of PVC stock suspension, a PE to PVC ratio of 1:1 was not achievable anymore

^e^Samples made in triplicates

^f^Nominal ratios based of light microscopy estimates

Samples 1–12, which did not include a density separation step, were prepared by first diluting respective volumes of stock suspensions (Table 1) in 8 mL of EtOH (samples 1–9) or SPT (samples 10–12) in glass vials. After vortexing and 20 s of ultrasound treatment, the suspensions were vacuum filtered on PC membranes. For samples 13 and 14, where a density separation step was included, the following procedure was applied: First, 4 mL of SPT solution was filled into centrifuge tubes (glass (DURAN), conical bottom, size 16 mm × 100 mm, volume 12 mL, VWR) and the required amount of stock suspensions (Table 1) were added to the tubes. Then the additional 4 mL of SPT solution was added. The suspensions were vortexed and then ultrasonicated for 20 s to mix the samples. For the density separation, centrifugation was performed for 2 h at 5908 RCF (Rotina 380, Hettich AG, Bäch, Switzerland). The supernatants of the centrifuged samples were carefully decanted into the glass funnel of the vacuum filtration unit. During decanting, the walls of the centrifuge tubes were rinsed with the rising solution.

### Simulations using Casino software

The software code Casino (v3.3.0.4) [71] was used to calculate the interaction of the electron beam with a 1-µm PS particle deposited on an Au-coated PC membrane. In addition, corresponding backscattered electron (BSE) images were calculated. Based on the results from the simulations, the optimal operational parameters for the SEM were derived in combination with a tailored Au coating thickness of the PC membranes.

### Scanning electron microscopy

The SEM (Gemini 460, Zeiss, Germany) was operated at acceleration voltages between 1 and 10 kV and currents ranging from 100 pA to 2 nA. A secondary electron (SE) detector (Everhard Thornley type detector) was used to image particle morphologies, and an in-lens BSE detector (energy-selective backscatter (EsB)) detector) was used for automated particle detection. For the EsB detector, a bias of 1500 eV was applied. For particle detection and elemental analysis, the software package AZtec-Feature (AZtec v6.0, Oxford Instruments, UK) was used. This software package allowed the automated detection of individual particles based on their backscattered electron signal (EsB detector) and reported the size of the detected particles as equivalent circular diameter. Automated elemental analysis of the detected particles was conducted using a windowless EDX detector (Ultim^Ⓡ^ Extreme, Oxford Instruments, UK), which, in combination with low acceleration voltages, allowed the detection of light elements (e.g., C, oxygen (O)) using K-lines and heavier elements (e.g., chlorine (Cl)) using L-lines. For the elemental analysis, the current of the electron beam was set to 500–800 pA, and a process time of “5” was selected, resulting in a detector dead time of ~ 15–25%. Spectra were recorded for 2 s for each particle.

For each PC membrane, randomly selected areas (7 × 5 individual images of 113 µm × 88.5 µm per area) were selected for automated particle analysis. After defining the respective areas, the process of image recording, particle detection, size classification, and elemental analysis of every individual particle was fully automated. The number of individual areas investigated per filter and related parameters is summarized in Table S3.

For the elemental quantification of the individual particles, C, nitrogen (N), O, fluorine (F), magnesium (Mg), aluminum (Al), silica (Si), and Cl were always included. In addition, an automatic identification routine was performed for every individual spectrum, and detected elements were also included in the quantification routine (built-in standardizations optimized for acceleration voltages of up to 5 kV). The Au signal resulting from the Au coating was only used for deconvolution, but Au was not quantified. The data of individual particles (size, elemental contents expressed in weight percent (wt%)) were imported into Matlab (R 2021a, The MathWorks, Inc.) for further processing. For all particles, a lower size limit of 1 µm (equivalent circular diameter) was set, whereas the upper size was set to either 10 µm or 100 µm.

### Raman microspectroscopy

Automated Raman microspectroscopic analysis including optical microscope image acquisition and Raman measurements was carried out using a WITec alpha300R system (Oxford Instruments, UK) equipped with a 100× objective lens (EC Epiplan-Neofluar, N.A. = 0.9, Carl Zeiss Microscopy GmbH, Germany). A 532-nm diode-pumped solid-state (DPSS) laser operating at 0.8 mW was used, with data collected with the setting “Optimize Fast” over a maximum of 40 accumulations at an integration time of 1 s each and a signal-to-noise ratio (SNR) limit of 25. Instrument control and automated image processing were managed via WITec Control SIX 6.2 software and the custom-developed open-source software TUM-ParticleTyper 2 [29, 72]. The Random Window Sampling technique was employed to cover a similar filter area as analyzed by SEM-EDX. It involved placing measurement windows of predefined size randomly across the filter surface to detect, identify, morphologically characterize, and quantify (microplastic) particles. For this analysis, the filter radius was limited to 7500 μm. The measurement window was 120 μm × 120 μm, with an active area of 105 μm × 105 μm to guarantee complete capture of each particle. Particles with a maximum diameter between 0.5 and 15 μm were included in the Raman measurements. The measurements for the filter area of 1.44 mm^2^ were done in triplicate. The spectral analysis was performed with WITec TrueMatch/ParticleScout 6.2 in the spectral range of 590 to 1770 cm^−1^ and 2690 to 3200 cm^−1^. Only particles with an High Quality Index (HQI) above 20 were considered as PE or PVC. The correct assignment of Raman spectra to PE and PVC was checked manually by the expert.

## Results and discussion

### Operational parameters of the SEM and optimization of sample substrates

#### Backscattered electron image contrast

**Fig. 1:**
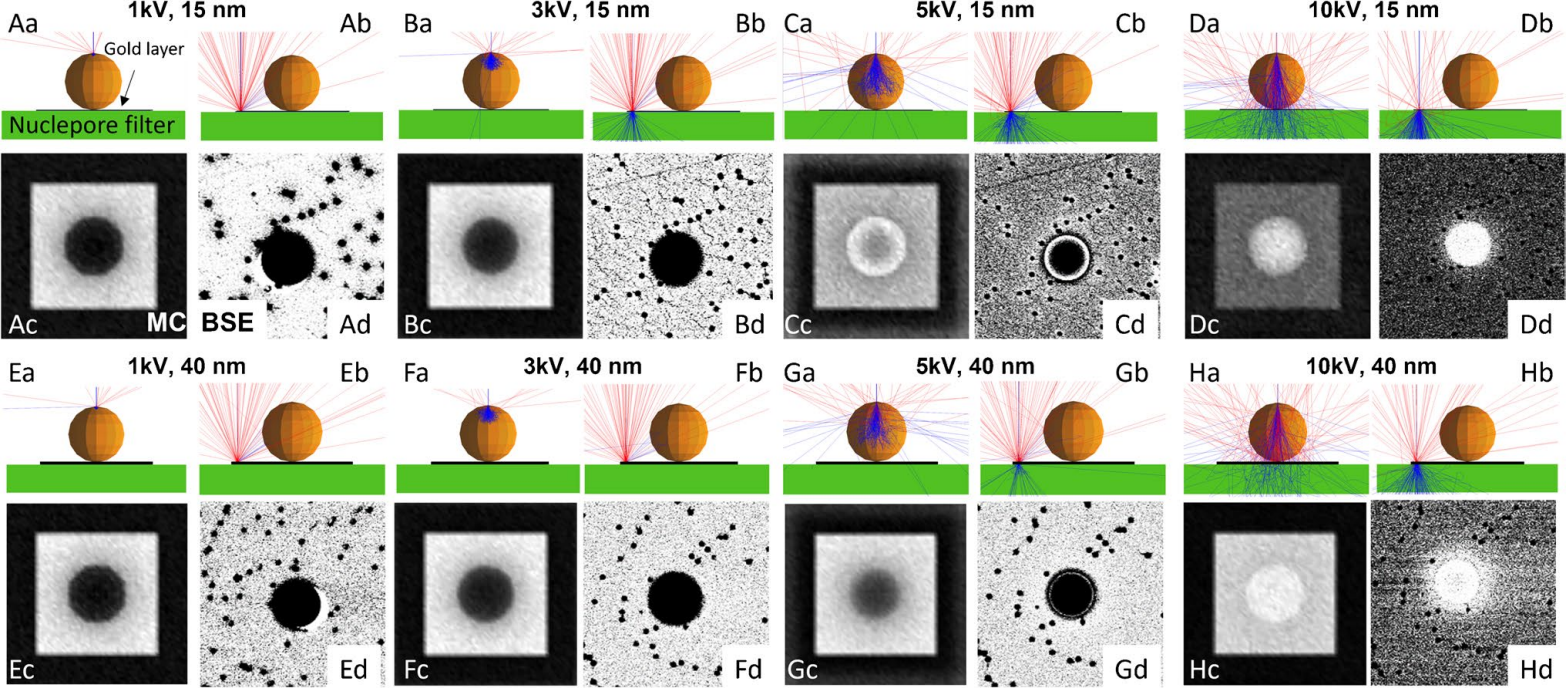
Interaction of the primary electron beam with either a 1 µm microplastic particle (polystyrene, orange; **A**–**H** (a)) or a thin gold layer (black horizontal bar, 15 nm (**A**–**D** (b)) or 40 nm (**E**–**H** (b))) on a polycarbonate (PC) membrane, derived from Monte Carlo (MC) simulations using the software code CASINO (v.3.3.0.4) [71]. The calculated (MC) and experimental backscattered electron (BSE) images of all interactions A–H are shown in their respective panels c and d. The simulations were performed for acceleration voltages of 1 (**A**, **E**), 3 (**B**, **F**), 5 (**C**, **G**), and 10 kV (**D**, **H**). Red: trajectories of the BSE. Blue: trajectories of the primary electrons. The small black dots in the BSE images correspond to the pores (0.1 µm) of the PC membrane

Polycarbonate membranes provide flat surfaces and are often used as substrates for particle imaging in electron microscopy studies. However, although these substrates are suited for morphological analysis of individual particles, the secondary electron signal representing the morphology of the particles is less well suited for an automated particle analysis, as such images are challenging to threshold (Fig. S3a). For automated particle detection, the BSE signal, representing a contrast in atomic number, is more suitable and therefore generally preferred for automated particle detection. However, as both the PC membrane and the MP particles are dominated by C, the resulting contrast based on backscattered electrons is very weak, hampering the automated detection of MPs. This can be overcome by coating the PC membranes with a thin layer of Au prior to depositing the MPs on the membranes. This enhances the contrast in atomic weight between the MPs and the underlying substrate. Deposited MPs, therefore, appear as dark areas on a bright background (Fig. S3b). Simulated BSE images obtained using the software code Casino (v.3.3.0.4) of an MP deposited on a PC membrane coated with 15 nm or 40 nm Au demonstrate the dependence of the BSE image contrast on the acceleration voltage and on the thickness of the Au coating (Fig. 1).

At an accelerating voltage of 1 and 3 kV, the interaction volume of the electron beam was considerably smaller compared to the volume of the PS sphere, and only a small amount of BSE was emitted from the PS particle (Fig. 1A, B, E, F (a)). This resulted in a strong negative contrast between the dark particle compared to the bright background of the 15 nm and the 40 nm Au coatings (Fig. 1A, B, E, F (c, d)), due to the higher BSE yield of Au compared to C. At 5 kV and 15 nm Au coating, the contrast started to reverse, and the edges of the PS particle appeared brighter than the background (Fig. 1C (c, d)). This was, on the one hand, caused by the increased production of BSE from the PS particle itself due to the larger interaction volume (Fig. 1C (a)), which was additionally cut towards the edge of the PS particle. On the other hand, at an acceleration voltage of 5 kV, a large portion of the primary electrons passed the 15 nm Au layer without producing BSE (Fig. 1C (b)) and the background therefore appeared darker compared to the images simulated at lower acceleration voltages (Fig. 1A, B (c)). At an accelerating voltage of 10 kV, the situation was further accentuated, and the contrast reversal was again increased (Fig. 1D (a–d)). Only at the lowest acceleration voltage of 1 kV was the 15 nm Au layer sufficient to entirely block the primary electron beam (Fig. 1A (b)). Already at 3 kV, a substantial fraction of the primary electrons penetrated the 15 nm Au layer and reached the underlying PC membrane (Fig. 1B (b)). These primary electrons interact with the PC membrane, resulting in the production of C x-rays, which may affect the classification of the MPs.

Increasing the coating thickness to 40 nm (Fig. 1E–H) did not substantially change the contrast of the simulated BSE images at acceleration voltages of 1–3 kV (Fig. 1E, F (c, d)), but the electrons were entirely absorbed by the 40 nm Au layer, also at 3 kV (Fig. 1F (b)). At 5 and 10 kV, the electron beam increasingly penetrated the 40 nm Au layer (Fig. 1G, H (b)), resulting in the same contrast reversal as already described for the 15 nm Au layer. At 10 kV, the contrast between the particle and the Au-coated filter was strongly reduced as the BSE signal was entirely dominated by the Au coating (underlying the PS particle; Fig. 1H (c, d)).

Based on these findings, the best BSE contrast is expected for accelerating voltages around and below 3 kV (Fig. 1A, B, E, F (c, d)). These results are transferable to all types of MPs since they are all essentially carbonaceous materials. For an acceleration voltage of 3 kV, an Au coating thickness of at least 40 nm is required to suppress production of C x-rays from the underlying PC membrane (Fig. 1F (b)).

Alternative substrates including adhesive carbon tape (e.g., [73]), TEM grids (e.g., [61, 66, 73]), or polished B crystals (e.g., [57, 74]) have been used to investigate airborne particles collected through impaction or electrostatic deposition. Although the latter two substrates provide low carbon (TEM grids) or even carbon-free (polished B crystals) backgrounds, quantitative deposition of particles from aqueous suspension is best achieved through filtration, which is incompatible with TEM grids or B crystals as substrates.

#### Carbon signal

The elemental analysis is essential to confirm that the particles detected based on the BSE signal are in fact MPs. Therefore, the electron beam should be blocked by the Au layer to limit additional C and O signals from the underlying PC membrane.

Based on the Monte Carlo simulations, the electrons from the primary beam are completely absorbed by the 40-nm Au layer at accelerating voltages below 3 kV, resulting in a C and O free EDX spectrum. Nevertheless, EDX spectra of Au-coated (40 nm) PC membranes recorded at accelerating voltages of 1 kV, 3 kV, 5 kV, and 10 kV demonstrate that the C and O signal intensities are greatly reduced at accelerating voltages of 1–3 kV but are not completely eliminated (Fig. S4). The most likely reason for this is beam-induced contamination resulting from poorly volatile organic molecules adsorbed on the Au surface. These molecules are mobilized and redeposited close to the electron beam during imaging and analysis [75]. This hypothesis is supported by the darkening of areas after imaging, as revealed by SE images (Fig. S5).

As the MPs are insulating materials, a coating of the samples was required to suppress electric charging of the MPs due to the electron beam during imaging and analysis. Although charging of the samples can be overcome by operating the instrument at variable pressures [76, 77], such conditions are not compatible with low accelerating voltages and windowless EDX detection systems. We, therefore, coated our samples with 5 nm of C. This was sufficient to avoid charging of the samples but also had an impact on the C signal intensities of the EDX spectra (Fig. S4a). The C counts increased from ~ 500 to about 1500 counts (under the experimental conditions). However, the signal intensities of C resulting from the C (and Au)-coated PC membrane were considerably smaller compared to the C signal of MPs (Fig. S4c). Based on these findings, the particles from the suspensions/supernatants were filtered on 40 nm Au-coated PC membranes (0.1 µm pore size, 25 mm diameter) and thereafter coated with a 5-nm layer of C.

#### Determination of particle size from SEM images

To assess whether these operational conditions also allowed an accurate size measurement, we deposited 1 µm PS MPs on Au-coated (40 nm) PC membranes and determined their size using the same operational conditions as were used for the experimental samples. The high intensity contrast between the MPs and the Au-coated PC membranes observed on the BSE images greatly facilitated automatic image segmentation. With a mean diameter of 1.1 µm for the PS spheres (Fig. S6), the results from the SEM analysis were in excellent agreement with the size provided by the manufacturer (1.06 µm). The particle size of C-based particles was generally underestimated by automated segmentation routines when using low-Z substrates (B crystals), most likely due to the poor contrast between B (*z* = 5) and C (*z* = 6) in BSE images [65, 74]. This underlines the suitability of our approach when targeting MPs, producing maximal contrast in BSE images between C (*z* = 6) and Au (*z* = 197).

### Particle identification and classification

**Fig. 2:**
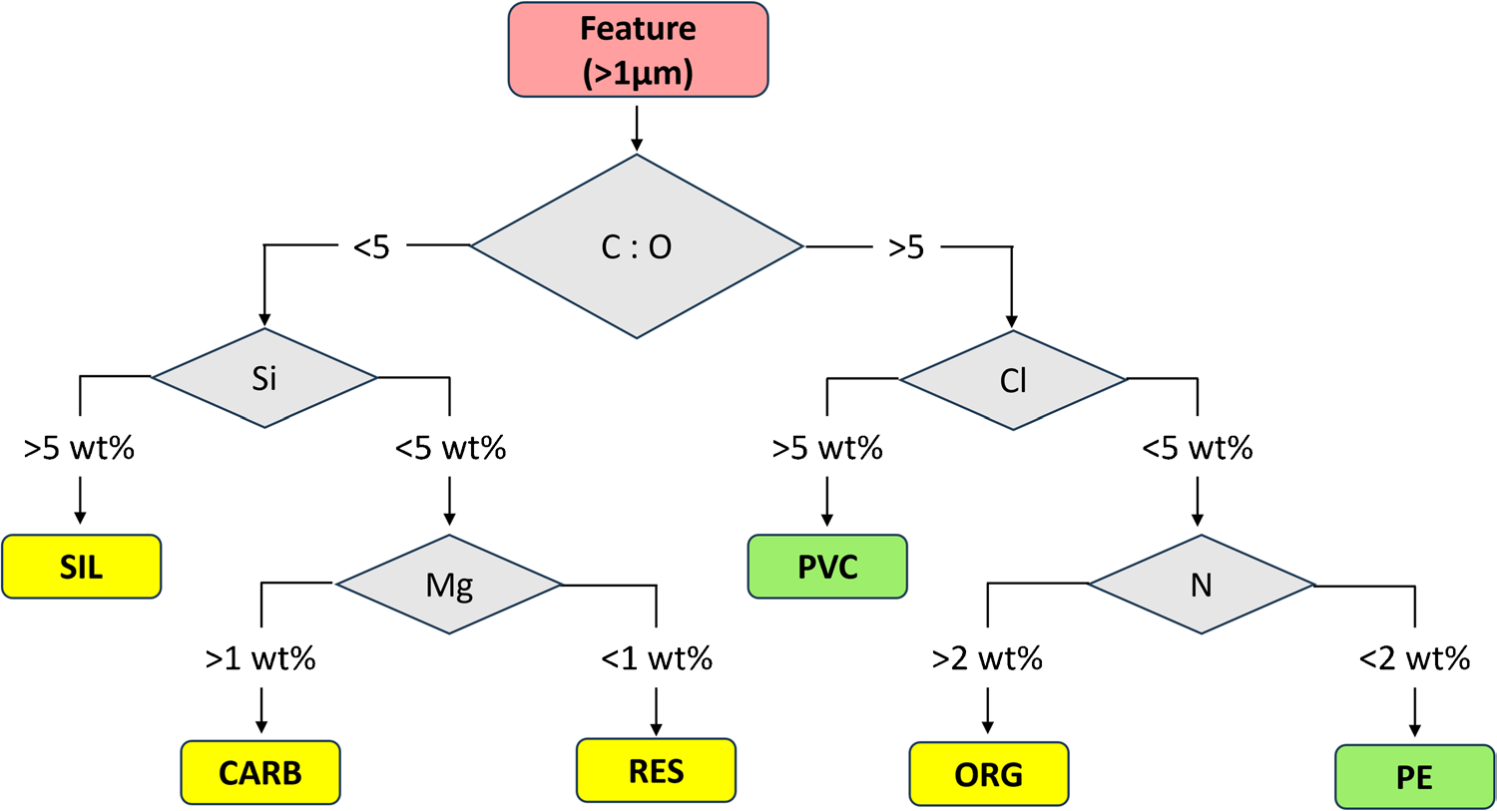
Classification scheme used to distinguish between polyethylene (PE), polyvinyl chloride (PVC), and soil particles represented by silicates (SIL), by means of energy-dispersive x-ray (EDX) analysis optimized for microplastic particles. The category “CARB” refers to a filler material that is characterized by the presence of magnesium (Mg) (see text for more details). The “ORG” category most likely represents natural organic material containing nitrogen. “RES” refers to particles that contained substantial amounts of oxygen but were low in silica and Mg. Si, silica; Cl, chlorine; N, nitrogen; C, carbon; O, oxygen

**Fig. 3:**
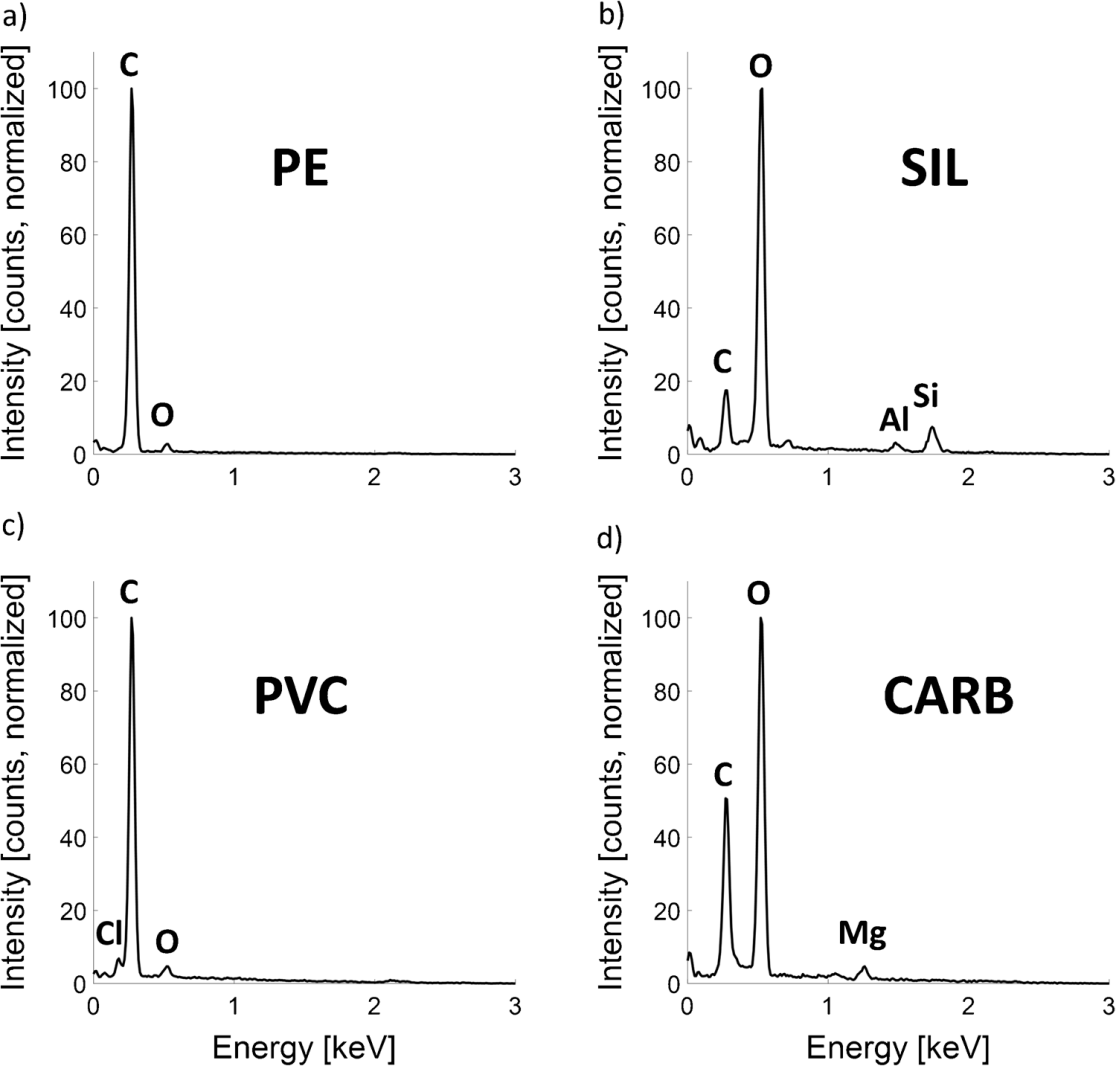
Energy-dispersive x-ray spectra of **a** polyethylene (PE), **b** polyvinyl chloride (PVC), **c** silicates (SIL), and **d** carbonates (CARB) obtained from individual particles. Samples were coated with 5 nm of carbon before analysis. Acceleration voltage: 3 kV

Based on the BSE image, representing a contrast in atomic number, MPs (and other particles) can be distinguished from the Au coating that appears brighter, due to the higher yield of BSE (Fig. S7). The identification of different particle types was achieved following the classification scheme given in Fig. 2, which is based on the elemental contents of individual particles obtained from EDX analysis. Typical spectra of the main particle categories are given in Fig. 3. Note that the exclusively C-H based polymers, such as PE, PS, and polypropylene (PP), cannot be distinguished based on EDX analyses, and we therefore consider PE as representative for all (exclusively) C-H based polymers.

Microplastic particles (PE and PVC) were characterized by C:O ratio > 5 and were separated from each other by the Cl signal. The EDX spectrum of PVC is characterized by a Cl peak (Cl Lα line) left of the C peak. Despite the high content of Cl in PVC (nominally 33 atom%, neglecting hydrogen (H) as H cannot be detected using EDX), its intensity is much lower compared to the intensity of the C peak. This is because the signal of Cl refers to an L-line and the signal from C refers to a K-line. The signal of N was used to distinguish between PE and the “ORG” particle category, the latter possibly representing particulate natural organic matter (Fig. 2). The EDX spectra of silicates (SIL), carbonates (CARB), and the residual particles (RES) were dominated by O (Fig. 3) and were therefore separated from the MPs by a C:O ratio < 5. To distinguish between SIL and CARB, the Si and the Mg contents were used (Fig. 2). Carbonate minerals were most likely present as calcite (CaCO_3_) and were related to filler materials in the PE that we used. However, under the experimental conditions applied, Ca-Kα (3.7 keV) was not accessible, and the Ca-Lα Line localized around 340 eV heavily interfered with C-Kα, making the quantification of Ca based on the Ca-Lα Line very challenging. Therefore, as the calcite in the filler material was always associated with Mg, we used Mg as a tracer. To confirm that the respective particles were calcite, several spectra of particles identified as CARB were additionally characterized at higher acceleration voltages. A spectrum recorded at an acceleration voltage of 7 kV is provided in Fig. S8 together with a quantification of the elemental composition. The stoichiometry of O:(Ca + Mg) = 3:1 fits well to the stoichiometry of (magnesium bearing)-calcite ((Ca,Mg)CO_3_). In addition to a substantial amount of O, the residual (“RES”) fraction was characterized by the lack (or very low contents) of Si and Mg. These particles therefore most likely represent organic particles with considerable amounts of O (e.g., cellulose), carbonates (without Mg), or oxides (i.e., Al oxides).

### Detection of microplastic (PE and PVC) and soil particles on Au-coated PC membranes

**Fig. 4:**
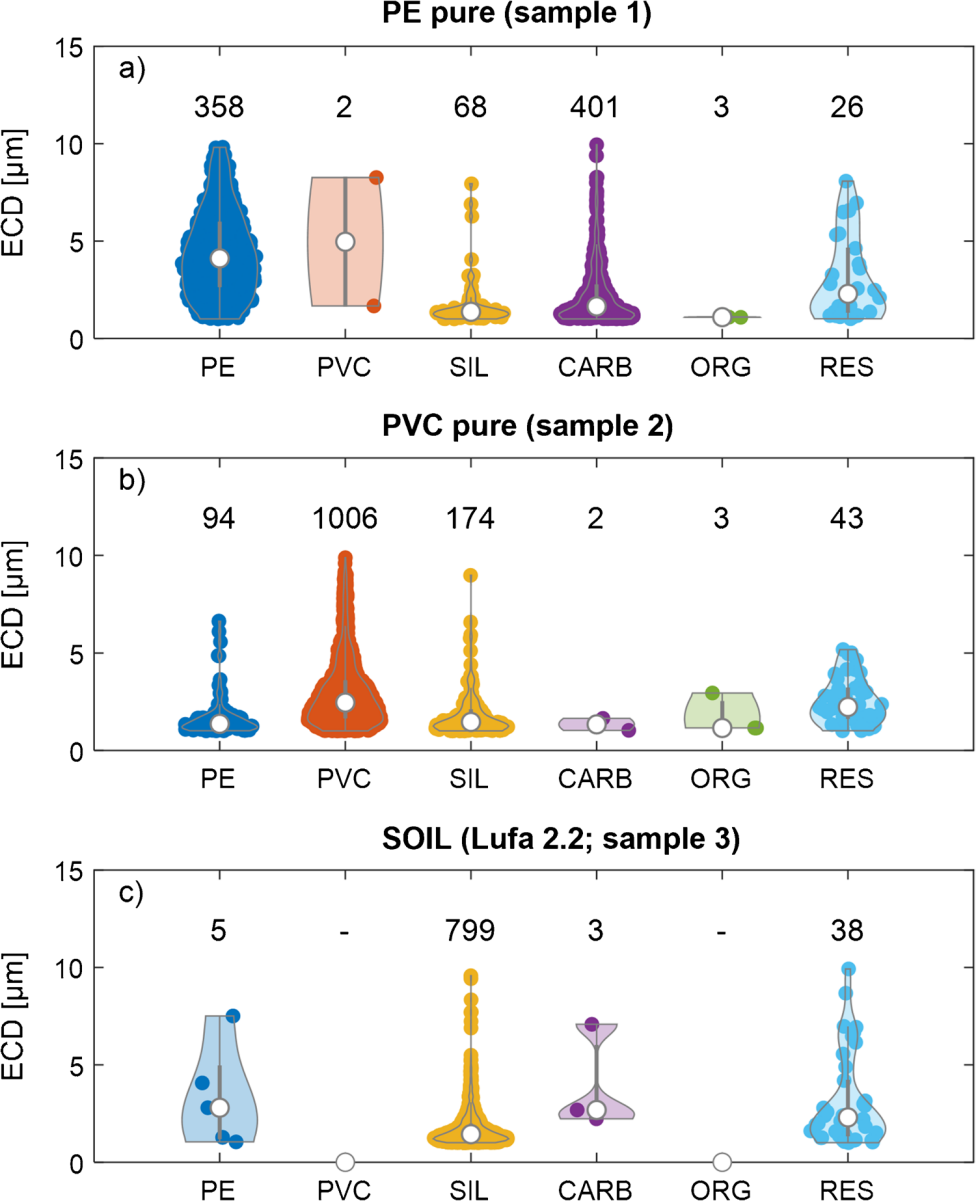
Violin plots of the particles detected in stock suspensions (samples 1–3) of (a) polyethylene (PE), (b) polyvinyl chloride (PVC), and (c) LUFA 2.2 soil filtered on gold-coated (40 nm) polycarbonate (PC) membranes. The PC membranes with the deposited particles were coated with an additional layer of carbon (5 nm) before analysis. “SIL” refers to silicate particles (which dominated the soil particles), “CARB” to carbonates, “ORG” to organic material containing nitrogen, and “RES” represents the residual particle category (as assigned in Fig. 2). Numbers on top of the violin plots refer to the number of particles of the corresponding category. A lower size threshold for the equivalent circular diameter (ECD) of 1 µm and an upper threshold of 10 µm was set for data analysis. Data with an upper size limit extended to 100 µm are provided in the supporting information (Table S3 and Fig. S10A). See Table 1 for a description of the individual samples.

To assess the capabilities of our approach to distinguish between PE (representing C-H only polymers) and PVC, particles from the respective stock suspensions were individually deposited on Au-coated (40 nm) PC membranes. The samples were investigated using SEM-EDX (Table 1, samples 1 and 2) after an additional C coating (5 nm) was applied. Four soils (LUFA 2.2, 2.4, 5M, and 6S) were prepared analogously (Table 1, samples 3–6), whereas the LUFA 2.2 soil was used to dilute the PE and the PVC particles in subsequent experiments. The other three soil samples were used to assess to what extent organic materials in the soil samples resulted in false positives. The SEM was operated using the operational parameters derived in the “Operational parameters of the SEM and optimization of sample substrates” section. The results are visualized as violin plots in Fig. 4 (1–10 µm) and Fig. S10A (1–100 µm) (Fig. S9 (1–10 µm) and S10B (1–100 µm) for the additional soil samples). Number concentrations of the MPs in the stock suspensions and in the mixtures, ratios of PE to PVC particles, and calculated recoveries are provided in Tables S4 (1–10 µm) and S5 (1–100 µm).

In sample 1 from the PE stock suspension, 358 PE particles were identified, translating into a particle number concentration of 3.4 × 10^5^ PE/mL. In the same sample, only 2 particles—out of 858 particles detected in total—were classified as PVC (Fig. 4a). In the PVC stock suspension (sample 2), 1006 PVC particles were identified, corresponding to a concentration of 2.3 × 10^6^ PVC/mL. Out of a total of 1322 particles, 94 particles were classified as PE in the PVC stock suspension (Fig. 4b). The higher amount of PE particles detected in the PVC stock suspension compared to the low number of PVC detected in the PE suspension likely resulted from the classification algorithm (Fig. 2) which relied on the detection of Cl for PVC, whereas PE was detected based on the absence of Cl and N, in addition to the C to O ratio. The smaller the particles, the more likely it is that the electron beam also hits the C-Au-coated PC membrane, which would increase the C content of the respective spectra at the cost of other elements, such as Cl and N. This hypothesis is supported by the particle size distribution (PSD) of PE in Fig. 4 and Fig. S10A; whereas the diameter of the PE particles detected on sample 1 (produced from the PE stock suspension) extends to > 10 µm and shows a maximum at around 3 µm, the PSD of the PE particles (erroneously) identified in sample 2 (produced from the PVC stock suspension) is skewed towards smaller particle sizes with the majority of the detected particles < 2 µm. We, therefore, assume that the PVC contents in experimental samples will be underestimated by as much as 10% and the PE contents will be overestimated accordingly. This uncertainty is likely to increase with decreasing particle sizes.

In sample 1, which was derived from the PE stock suspension made in EtOH, substantial amounts of carbonate minerals (“CARB” particle category) were additionally observed (Fig. 4a and S10A, C), which most likely represented filler materials of the respective raw (PE) material, as discussed in the “Particle identification and classification” section. The PSD of the CARB particles was heavily skewed towards smaller particle sizes, with most of the particles being around 1 µm. Although present in considerable amounts, the particles classified as CARB (Fig. 4a and S10) were clearly distinguishable from the PE, PVC, and SIL particles and did thus not compromise the detection of PE and PVC particles.

Particles from soil stock suspensions (samples 3–6) were dominantly classified as SIL and only 0–5 particles were classified as PE and 0–3 as PVC (Fig. 4c, S9 and S10B). These MPs contributed to less than 0.6% of the total amount of particles detected. Their origin was, therefore, not further investigated. Furthermore, particles of the categories (CARB and ORG) were only detected in small amounts (< 2%). In the soil samples LUFA 2.4, 5M, and 6S, substantial amounts of particles were assigned to the “RES” category (as defined in Fig. 2). These particles were most likely organic particles (e.g., cellulose) which, due to their considerable O content, were classified as residual (“RES”). These results demonstrate that our classification algorithm is robust towards false positives of PE and PVC from a diverse set of soil samples, and an additional digestion step (e.g., oxidative digestion) does not seem to be necessary for such (soil) matrices.

In aerosol samples, “carbon-rich” and soot particles were identified using SEM-EDX analysis. The elemental composition of these particle types was dominated by C with small amounts of O [64, 70]. Such spectra would most likely be classified as PE by our algorithm. Combustion-related particles (e.g., soot), however, are typically found in the sub-micron size range [65, 78–80]) and would therefore not be detected by our protocol. Furthermore, biological particles (e.g., spores of fungi) were identified as an important particle fraction in the supramicron size range [78, 81]. The elemental composition of these particles was also dominated by C. Biological particles contain substantial amounts of O and would, thus, most likely end up in the residual particle category by our classification algorithm.

Significant amounts of SIL particles were identified in the stock suspension of both PE and PVC (Fig. 4a, b). The PSD of the SIL particles detected in all samples was very similar and skewed towards smaller particle sizes. Furthermore, manual inspection of individual EDX spectra always showed a signal from Si-Kα, suggesting that the SIL particles observed on filtered PE and PVC stock suspension were related to impurities in the (pristine) polymer powders or were introduced during the preparation of the stock suspension from the respective powders.

### Identification of microplastic particles (PE and PVC) in the presence of soil particles

**Fig. 5:**
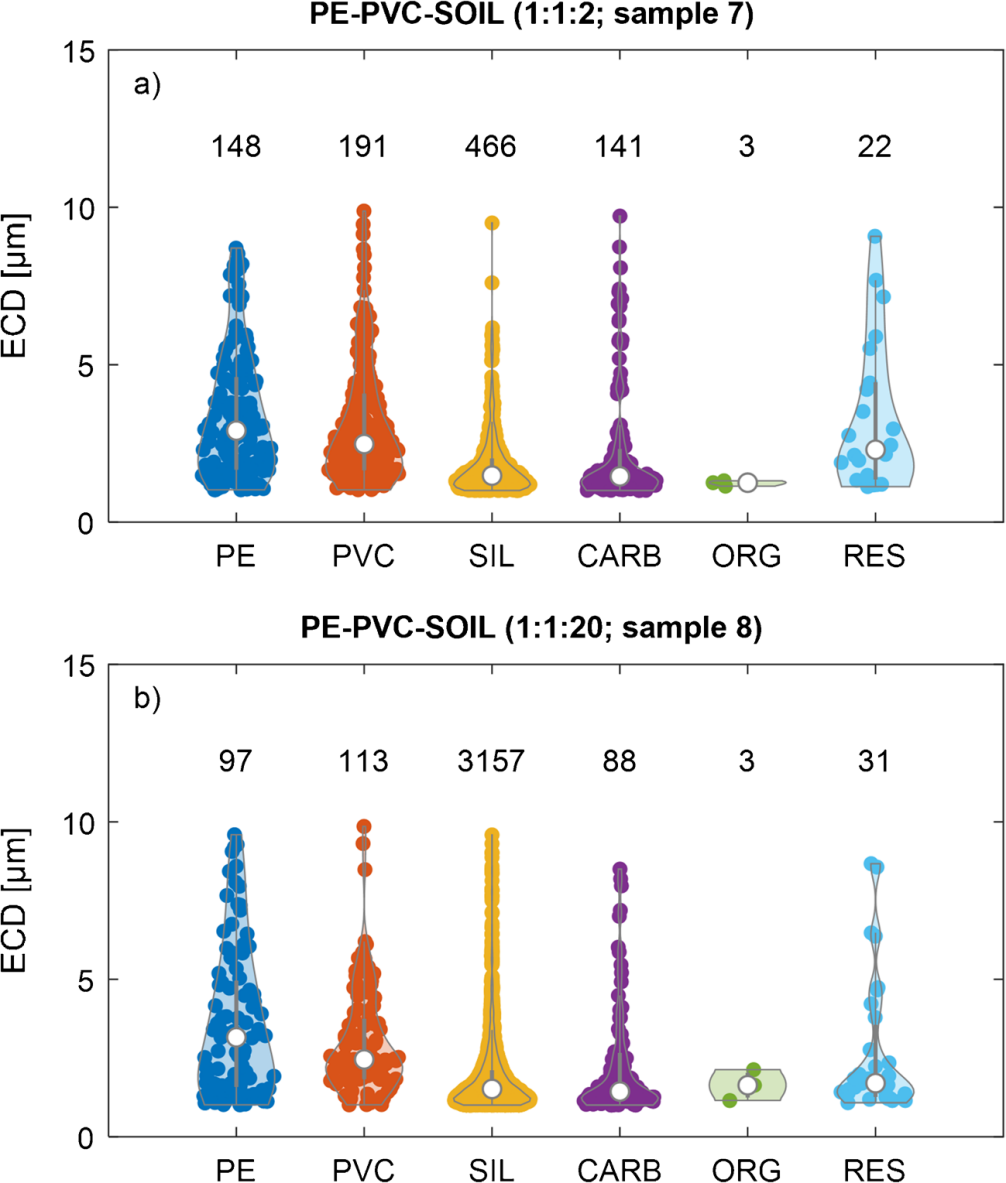
Violin plots of the particles detected in dilutions of stock suspensions of polyethylene (PE) and polyvinyl chloride (PVC) in LUFA 2.2 soil, at (nominal) ratios of 1:1:2 (a, sample 7) and 1:1:20 (b, sample 8), respectively. The suspensions were filtered on gold-coated polycarbonate (PC) membranes. The PC membranes with the deposited particles were coated with a 5-nm carbon layer before analysis. “SIL” refers to silicate particles (which dominated the soil particles), “CARB” to carbonates, “ORG” to organic material containing nitrogen, and “RES” to residual particle category (as assigned in Fig. 2). Numbers on top of the violin plots refer to the number of particles of the corresponding category. A lower size threshold for the equivalent circular diameter (ECD) of 1 µm and an upper threshold of 10 µm was set for data analysis. Data with an upper size limit extended to 100 µm are provided in the supporting information (Table S3 and Fig. S10C). See Table 1 for a description of the individual samples.

To assess the dynamic range of the method, PE, PVC, and soil particles were mixed at (nominal) ratios of 1:1:2 (Table 1, sample 7) and 1:1:20 (Table 1, sample 8). Violin plots of the data from these two samples are provided in Fig. 5 and Fig. S10C.

The observed recoveries for PE particles were between 80 and 120% and for PVC between 60 and 100% (Tables S4 and S5). The generally lower recoveries for PVC compared to PE may result from different surface properties and related aggregation behavior of the PVC compared to the PE particles, as reported by [82, 83], but may also result from slight variations in the mixing and pipetting of the MP stock suspensions. Furthermore, it has to be considered that up to 10% of the PVC particles are likely to be classified as PE particles (see the “Detection of microplastic (PE and PVC) and soil particles on Au-coated PC membranes” section).

For the 1–10 µm size fraction, the (measured) particle number–based ratios of PE to PVC in samples 7 (Fig. 5a) and 8 (Fig. 5b), both with the same nominal ratio of 1, were 0.77 and 0.86. The (expected) ratios calculated based on the actual stock concentrations were 0.59 (Table S4). Similar trends were also observed when including all MPs (1–100 µm, Table S5). The ratio between the PE and the SIL particles was measured at 0.32, in good agreement with the expected value (calculated based on the particle number concentration in the stock suspensions in combination with the respective volumes used for the mixtures, Table S4) of 0.22. Also, the measured PVC to SIL ratio (0.41) was in good agreement with the expected ratio (0.38, Table S4). The substantial deviation of the expected (and measured) (PE, PVC) to SIL ratios from the nominal ratio of 1 most likely results from the small particle size of the SIL particles, which were only poorly detected in the optical microscope (data from the optical microscope served as a basis to calculate the nominal ratios). A tenfold dilution in the SIL suspension very consistently resulted in a tenfold smaller ratio of PE to SIL (0.03) and of PVC to SIL (0.04), suggesting limited interaction between MPs and SIL particles (Table S4). Note that the ratio between PE and CARB particles was close to 1 in both samples (samples 7 and 8, Fig. 5) as was observed for the PE stock suspension (sample 1) (Fig. 4a), supporting our hypothesis that the CARB particles were related to filler materials in the PE and already present in the respective stock suspension.

Based on these findings, we concluded that a ratio of MPs to SIL of 1:10 still allows for reliable detection of MPs. This, however, will require substantial enrichment of MPs from soil matrices, where MPs are expected in low or even trace concentrations. We therefore evaluated the potential of a density separation step to enrich the MPs. The results are discussed in the following sections.

### Recovery of MP particles (PE, PVC) spiked to soil matrices

To determine the recovery of MPs from soil matrices, and to assess the suitability of a density separation approach to sufficiently separate MPs from soil matrices and enrich MPs in the supernatant, stock suspensions of PE, PVC, and soil (LUFA 2.2) were individually prepared in SPT solutions and then mixed. We conducted three different experiments, always using the same volume of PE and PVC stock suspensions (samples 10–14) and additionally varying the volume of the soil suspension (sample 14). Therefore, the number of the PE and PVC particles present was the same in all three experiments. The goals of these experiments were to evaluate (i) the variability of the particle loadings on the PC membranes resulting from the filtration step, (ii) the impact of the density separation procedure on the MP recovery, and (iii) the impact of the presence of soil particles during the density separation on the recovery of the MPs. The actual particle number concentrations calculated based on the number of particles detected on the individual filters, recoveries for PE, PVC, and SIL particles, as well as ratios between PE, PVC, and SIL particles, are provided in Tables S4 (1–10 µm) and S5 (1–100 µm). The recoveries reported for samples 10–14 were calculated relative to the mean particle number concentration determined for samples 10–12 (mixtures directly filtered without density separation).

#### Production of reference samples and variability of particle loading

Samples 10–12 were prepared by mixing PE, PVC, and soil stock suspensions (nominal ratio: 1:1:2) and directly filtering this mixture on Au-coated PC membranes without any additional treatment. These triplicate samples allowed assessing the variability associated with sample processing (pipetting and filtration steps). Violin plots of the individual triplicate samples showed very similar particle numbers (Fig. S10D (1–100 µm) and Fig. S11 (1–10 µm)) translating into particle number concentrations of 2.60–2.84 × 10^5^ #/mL for PE, 2.91–3.53 × 10^5^ #/mL for PVC, and 2.39–2.92 × 10^7^ #/mL for SIL particles (Table S4: 1–10 µm; Table S5: 1–100 µm). The relative standard deviation of the total number of detected particles in the respective category (1 × σ) ranged from ~ 5% (PE) to ~ 10% (PVC and SIL). Thus, the uncertainty of the particle number related to the sample preparation (excluding density separation) can be estimated to around 10%. The measured ratios between different particle categories were as follows: PE-PVC, 0.97–1.07; PE-SIL, 1.12–1.31; and PVC-SIL, 1.16–1.28 (Table S4).

#### Impact of density separation on the recovery of microplastic particles

**Fig. 6:**
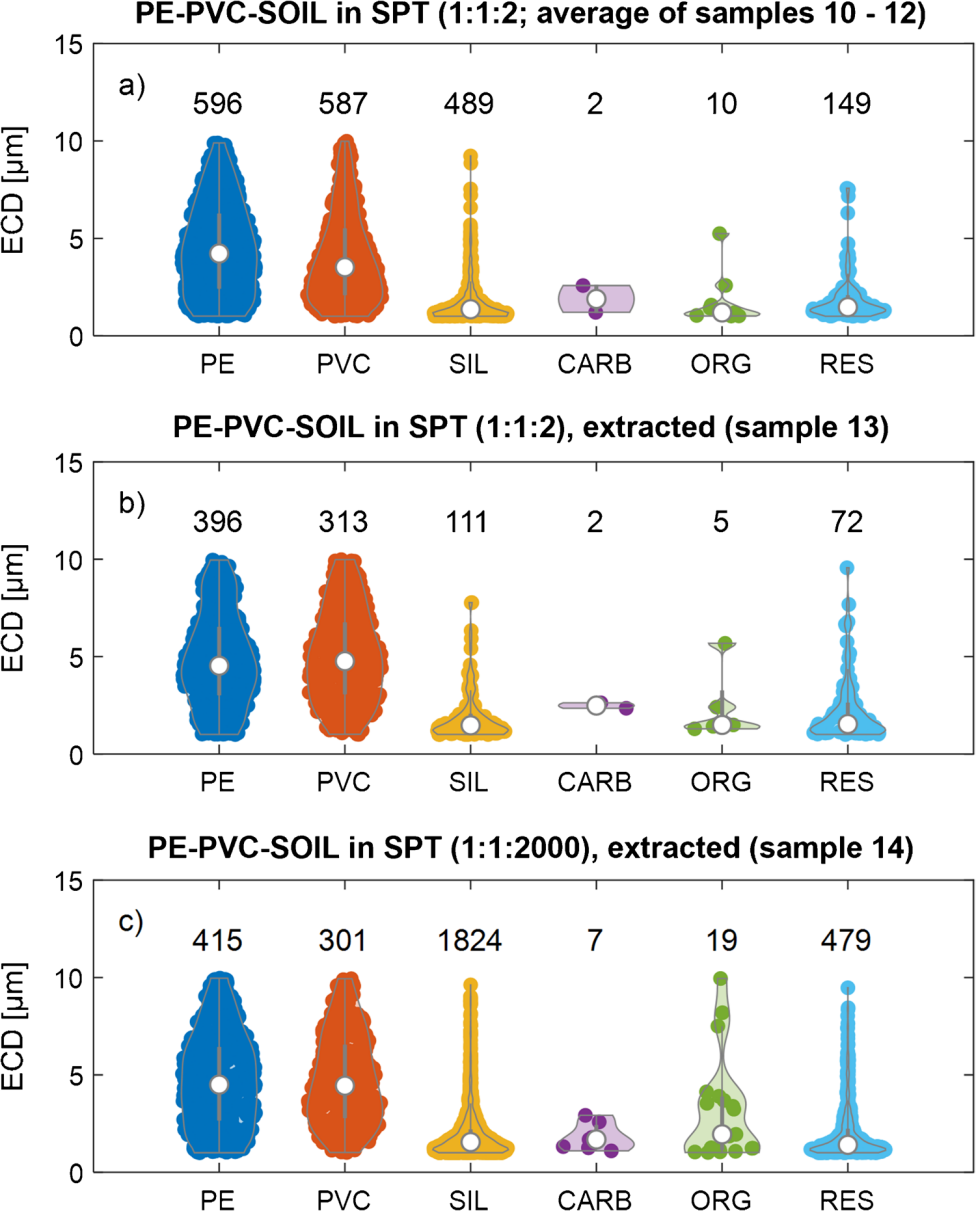
Violin plots of the particles detected in mixtures of polyethylene (PE), polyvinyl chloride (PVC), and soil stock suspensions (ratio: 1:1:2) (a) directly filtered on gold (Au) coated polycarbonate (PC) membranes (average of samples 10–12), (b) density separated and then filtered on Au-coated PC membranes (sample 13), and (c) diluted 1:1000 in a soil stock suspension, density separated and filtered on Au-coated PC membranes (sample 14). The PC membranes with the deposited particles were coated with a 5-nm carbon layer before analysis. “SIL” refers to silicate particles (which dominated the soil particles), “CARB” to carbonates, “ORG” to organic material containing nitrogen, and “RES” to residual particle category (as assigned in Fig. 2). Numbers on top of the violin plots refer to the number of particles of the corresponding category. A lower size threshold for the equivalent circular diameter (ECD) of 1 µm and an upper threshold of 10 µm was set for data analysis. Data with an upper size limit extended to 100 µm are provided in the supporting information (Table S3 and Fig. S10E). See Table 1 for a description of the individual samples

To investigate the impact of the density separation procedure on the recovery of PE and PVC particles, a PC membrane was prepared by mixing the same volumes of PE, PVC, and soil stock suspension as in samples 10–12 and extracting the MPs from this mixture using density separation (sample 13, Table 1). Violin plots of the identified particles are provided in Fig. 6b and Fig. S10Eb.

The recoveries for PE and PVC were 66% and 53%, respectively (Table S4 (1–10 µm) and S5 (1–100 µm)). Considering an estimated uncertainty of around 10% for the depositional heterogeneity (derived from triplicate experiments described above), the density separation still led to a significant loss of PE and PVC particles. The comparison between the violin plots for the stocks directly filtered (Fig. 6a) to the violin plots for the stock suspension after density separation (Fig. 6b) showed that the losses were dominated by the smallest particle sizes. This loss may have occurred during the sample preparation, for example, due to the attachment of the MP particles to the walls of the glassware. Alternatively, the formation of agglomerates between individual MP particles (PE-PE and PVC-PVC homoagglomerates and PE-PVC heteroagglomerates) would also result in an apparent particle loss and would lead to a shift of the PSD towards larger particles. A direct comparison of the PSD of the PE and PVC particles before and after the density separation process indeed shows a broadening of the PSD of both polymer types after density separation (Fig. S12). These observations are in line with results from a recent study where the efficient formation of heteroagglomerates between MPs and silanized (hydrophobic) magnetite was explained by hydrophobic interactions. Note that also in [82], the presence of additional (hydrophilic) particles (cellulose in that case) did not affect the formation of heteroagglomerates between MPs and silanized magnetite. It is, thus, possible that the “apparent” loss of the smallest MPs indeed reflected the formation of (hetero- or homo-)agglomerates between individual MPs and that the presence of (hydrophilic) silicate particles only marginally impacted this process. The trend of decreasing recoveries with decreasing MP size (Fig. S12) is consistent with results from a study where fluorescently labelled PS spheres (1–5 µm) were spiked into a soil matrix and separated by density separation. The extraction efficiencies derived from the bulk fluorescent signal dropped with decreasing size of the PS spheres and reached ~ 30% for the 1-µm spheres [84].

#### Impact of high concentrations of soil particles on the microplastic particle recovery

To investigate whether soil particles represented by the silicates (SIL particle category) present at much higher number concentrations compared to MPs affect the recovery of MPs and whether the density separation process sufficiently enriched the MPs, a PC membrane was prepared by diluting the same volumes of PE and PVC stock suspensions (as in the two previous experiments) in a soil stock suspension by a factor of 1000. This mixture was density separated and filtered on an Au-coated PC membrane (sample 14 in Table 1). Violin plots of the detected particles are provided in Fig. 6c (and Fig. S10Ec).

Despite the overwhelming presence of SIL particles, the recoveries of the PE and the PVC particles remained at 70% and 50% (Table S4 (1–10 µm) and S5 (1–100 µm)), respectively, as observed for experiments conducted at PE to PVC to SIL (nominal) ratios of 1:1:2 (sample 13, section “Impact of density separation on the recovery of microplastic particles”). As discussed above, only limited interaction/agglomeration occurred between the MPs (PE and PVC) and the SIL particles, leading to an efficient and selective separation of MPs from the soil matrix. Furthermore, the PSD of the recovered PE and PVC particles was very similar to the PSD of the PE and PVC particles from the 1:1:2 (nominal) mixture after density separation (sample 13) (Fig. 6b, c). Considering the relative uncertainties for the particle loadings of ~10% (derived from the triplicate samples 10–12) and assuming the relative uncertainties remain the same also for samples 13 and 14, this translates into a relative uncertainty of ~20% for the recoveries, or 70% ± 14% for PE and 50% ± 10% for PVC.

The soil particles were efficiently removed from the matrix during the density separation process. The volume of the stock suspension used to prepare this sample (number 14, Table 1) was 1000× the volume that was used to prepare the sample numbers 10–12. As on average 500 SIL particles were detected on samples 10–12, around 500,000 SIL particles would be expected on sample 14 (without density separation). After the density separation process, however, only ~ 2000 SIL particles were observed, which translates into a removal efficiency of 99.6% (recovery of 0.4%, Table S4). Furthermore, the ratio between MPs and SIL particles increased from (expected) 0.1 to 23% for PE and 17% for PVC, which is well above our 10% limit (see the “Identification of microplastic particles (PE and PVC) in the presence of soil particles” section), resulting in samples well-suited for automated SEM-EDX analysis.

### Comparison between SEM-EDX and Raman measurements

The SEM-EDX data are element-specific, which allowed us to identify the targeted MPs, but they do not provide any information about the chemical structure of the polymers. To evaluate to what extent our element (ratio)-based classification scheme corresponds to the results obtained from a structural classification, we conducted additional measurements using Raman microspectroscopy. For that purpose, filters containing PE, PVC, and SIL particles (nominal ratio of 2:1:3, sample 9) were prepared in duplicates, whereas one replicate was investigated using automated SEM-EDX and the other replicate was investigated using automated Raman microspectroscopy. Results are provided in Table S6. Triplicate Raman measurements resulted in 285 (± 26, 1σ) PE particles per mm^2^, whereas the SEM data resulted in 303 particles per mm^2^. Results for triplicate filters measured with the SEM showed a relative standard deviation of ~ 10% (Figure S11) which is similar to the 9% calculated for the Raman measurements. Considering these uncertainties of around 10%, there is an excellent match between the SEM-EDX and the Raman quantification of the PE particles (recovery of Raman relative to SEM-EDX of 84–100%, Table S6). For the PVC particles, 165 (± 7) particles/mm^2^ were detected by Raman, whereas SEM-EDX measurements resulted in 337 particles/mm^2^. This difference of around a factor of 2 (recovery of Raman relative to SEM-EDX of 47–51%, Table S6) cannot be explained by statistical variations of the PVC particles on the filter. The lower apparent abundance of PVC particles may be due to their relatively few distinct Raman signals (compared to PE and other polymers, like PP, PS, polyethylene terephthalate (PET)), low overall signal intensity, and frequent fluorescence interference. These factors hamper spectral identification, often resulting in low spectral quality and, hence, low HQI values, potentially leading to an underestimation of PVC particles due to an increased number of false negatives. Additionally, the laser power had to be limited to a very low value (0.8 mW), to avoid structural degradation of the small-sized (PVC) particles due to absorption of green light (532 nm laser) by Au coating of the PC membrane. Furthermore, the Au-coated filter showed a weak fluorescence background, potentially masking the weak signal from the PVC particles. Therefore, alternative coating, e.g., Al, is recommended for the Raman analysis of MPs in the lower µm range [30]. Thus, Au-coated PC membranes being ideal for SEM-EDX analysis of MPs were less suitable for automated Raman analysis of small-sized MPs [30], underlining the importance of tailoring sample carriers to the respective analytical method.

## Conclusions

Applying a gold (Au) coating on polycarbonate (PC) membranes solves the challenges associated with low atomic weight contrast between microplastic particles (MPs) and underlying PC membranes, which are the prime choice for separating MPs from liquid matrices through filtration. This setup, however, requires optimizing operational parameters of the scanning electron microscope (SEM) for imaging and elemental analysis. Based on Monte Carlo simulations, an acceleration voltage of 3 kV and PC membranes coated with at least 40 nm of Au appear most suitable. Applying these conditions, the backscattered electron (BSE) signal provides a high-contrast image—dark MPs on a bright (Au) background—that greatly simplifies the automated detection of individual (microplastic) particles. The use of a windowless energy-dispersive x-ray (EDX) analysis system further allows distinguishing between different (microplastic) particle types based on elemental ratios of light elements, as demonstrated for polyethylene (PE)—representative for polymers exclusively containing carbon and hydrogen in their structure—and polyvinyl chloride (PVC) particles.

In soils and probably also other (natural) matrices, environmental (silicate) particles exceed MPs by several orders of magnitude, calling for an efficient enrichment of the MPs before analysis. By applying a density separation step, the MPs were successfully enriched by a factor of ~150 (190 for PE, 140 for PVC) from a 1 (MPs):1000 (soil) mixture and reached a number-based fraction of ~20%. This is well within the analytical window of automated SEM-EDX analysis for MP detection, making our setup very promising for analyzing small MPs in soil matrices. Particle-based recoveries were between 50 and 70% for PVC and PE, respectively. Particle losses most likely represent real losses to the glassware and apparent particle losses through the formation of (homo)agglomerates. It is worth pointing out that these recoveries are not specific to the electron microscopy approaches as presented in this study but are rather inherent to the applied sample preparation protocol. This is also supported by comparative Raman measurements, where very similar data for PE particles were obtained. Environmental weathering of MPs, such as exposure to UV-light and resulting photooxidation of the MP surfaces, may distort the elemental ratios of the pristine polymers. Whether the modified elemental ratios would interfere with the established classification algorithm and, if so, to what extent the classification scheme would have to be adapted to account for environmentally weathered MPs will need to be addressed in future studies.

## Supplementary Information

Below is the link to the electronic supplementary material.

Supplementary Material 1 (PDF 1.63 MB)

## Data Availability

The datasets generated during this study are available from the corresponding author upon reasonable request.
