# Supplementary material for: Quantification of small (1–10 µm) microplastic particles in soil matrices using automated scanning electron microscopy: possibilities and limitations
Source: Anal Bioanal Chem. 2025 Oct 14;417(27):6191–208. doi: 10.1007/s00216-025-06111-8 (PMC12583325; doi:10.1007/s00216-025-06111-8)
Supplement: Supplementary file 1 — Supplementary Material 1 (PDF 1.63 MB) [file 216_2025_6111_MOESM1_ESM.pdf]

Supporting Information

Quantification of Small (1-10µm) Microplastic Particles in Soil Matrices Using Automated Scanning Electron Microscopy: Possibilities and Limitations

Ralf Kaegi[1*], Matthias Philipp[1], Isabel S. Jüngling[2], Natalia P. Ivleva[2] and Thomas D. Bucheli[3]

1: Eawag, Swiss Federal Institute of Aquatic Science and Technology, Überlandstrasse 133, CH-8600 Dübendorf

2: Chair of Analytical Chemistry and Water Chemistry, Institute of Water Chemistry (IWC), TUM School of Natural Sciences (NAT, Dep. Chemistry), Technical University of Munich, Lichtenbergstraße 4, D-85748 Garching

3: Agroscope Environmental Analytics, 8046 Zurich, Switzerland

* Corresponding author. Tel.: +41 (0)58 765 52 73; e-mail: ralf.kaegi@eawag.ch.

The supporting information contains one section on the characterization of polyethylene (PE) and polyvinyl chloride (PVC) stock suspensions (S1), one section on of particle losses during filtration and filter handling (S2), 12 Figures and 6 Tables.

S1: Characterization of PE and PVC from stock suspensions

To obtain FT-IR spectra of the microplastic particles (MPs) from polyethylene (PE) and polyvinyl chloride (PVC) stock suspensions ~5mL of either stock suspension were filtered (Polyethersulfon filter, 0.22µm pore diameter, 47mm diameter, Merck, Germany), the retained MPs were transferred to a glass slide and compressed into a central pile. Attenuated total reflection Fourier Transform – Infrarot (FT-IR) spectra (Fig. S1) of these MP powders showed the characteristic absorption peaks of PE and PVC (Table S1). In addition to the suspensions, the source material of the PE suspensions was also available as pellets. Individual pellets of ~ 3mm diameter were investigated using µ-x-ray fluorescence spectroscopy (µ-XRF) for the presence of elements related to filler materials. Energy dispersive x-ray fluorescence spectra of individual pellets indeed showed a minor peak of calcium (Ca) (Fig. S2), which probably was related to a filler material. This issue will be further discussed based on electron microscopy data presented in the main manuscript (section 4.2).

S2: Quantification of (apparent) particle losses associated with filtration, filter handling and particle analysis.

To evaluate to what extent MPs were lost during the filtration step, 4.5mL of a PS (3µm diameter) suspension with a certified particle number concentration (23'140#/mL ±10%, Count Check Beads) were filtered on gold (Au) coated PC membranes. The particle numbers were evaluated on recorded images. Results from duplicate experiments demonstrated a recovery of 87 and 88% (Table S2). Considering the uncertainties associated with the number concentration in the certified suspension of

±10% and additional uncertainties related to the extrapolation of the investigated areas to the whole filter area, a correction of possible particle losses associated with the filtration, filter handling and particle detection is not justified. Therefore, reported particle number concentrations (c) of MPs in suspensions are calculated following eq. (1).

$$c\left[\frac{\#}{mL}\right] = \frac{N_{det}[\#]}{V\,[mL]} \cdot \frac{A_{tot}[\mu m^2]}{A_i[\mu m^2]} \qquad \text{eq. (1)}$$

Whereas N_{det}: number of MPs detected, A_i: filter area investigated, A_{tot}: total filter area, and V: filtered volume.

Figures:

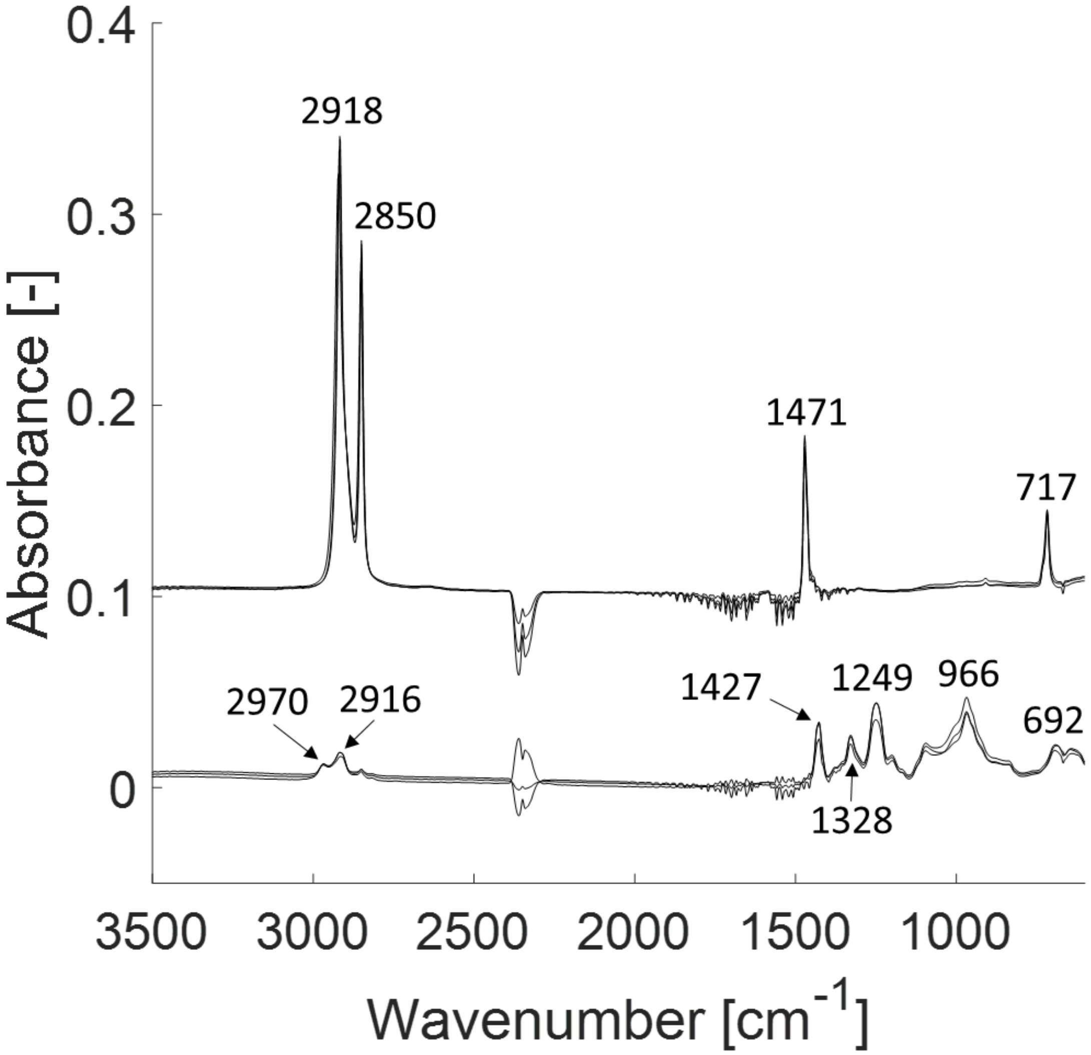

Fig. S1 Attenuated total reflection (ATR) Fourier transform - infrared (FT-IR) absorption spectra of the polyethylene (PE) and polyvinyl chloride (PVC) particles. Stock suspensions were filtered, and microplastic particles transferred to glass slides. ATR measurements were then conducted on dried microplastic powders. Assignments of the indicated peaks are provided in Table S1. For ATR measurements, a µ-FT-IR system (Cary 670 FTIR instrument, Cary 610 IR microscope, Agilent) was used. Measurements were performed from 4000 to 400 cm^{-1} at a spectral resolution of 4 cm^{-1}. Spectra from the background and from the microplastic powders were integrated 64 times.

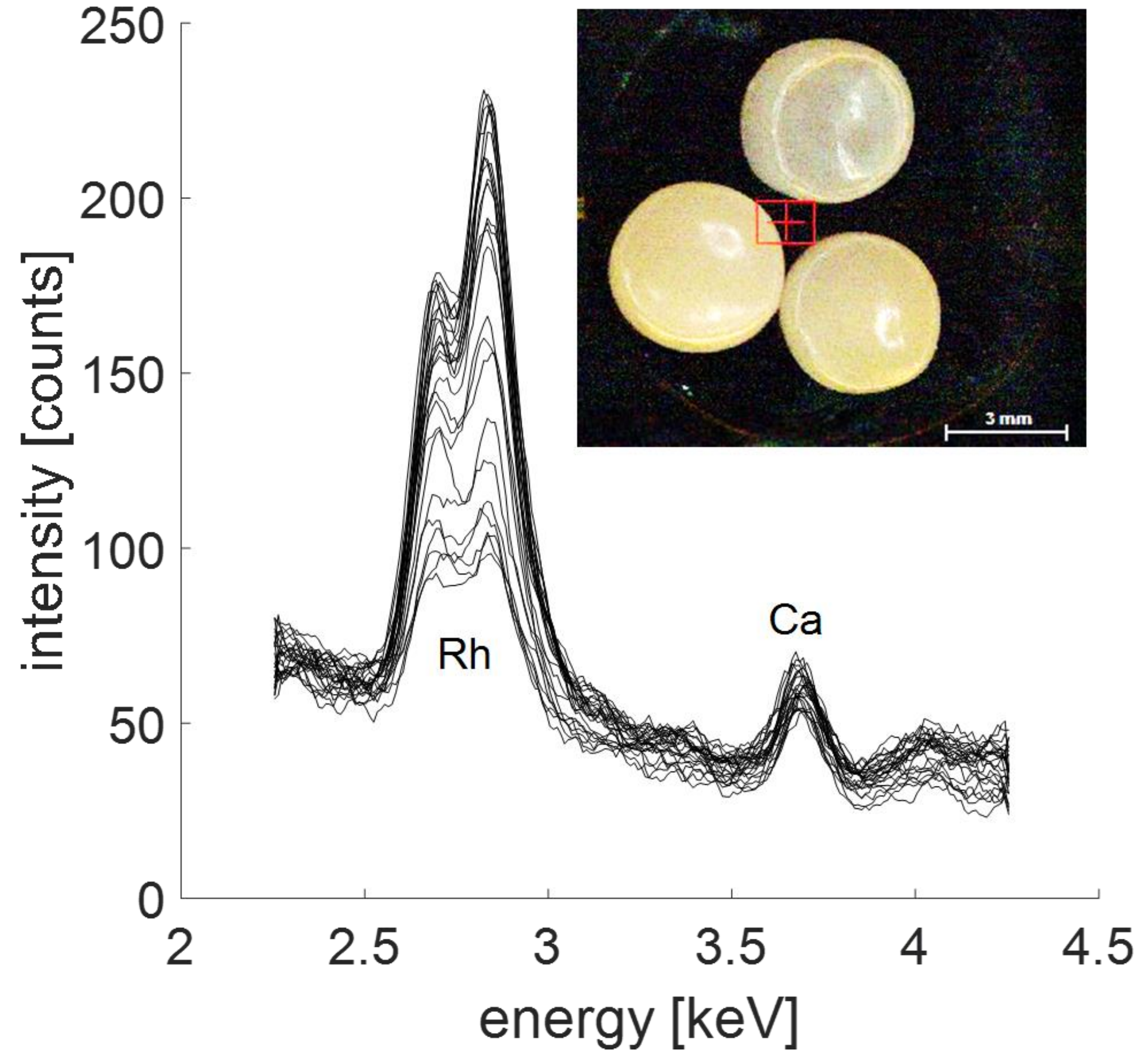

Fig. S2 Twenty-five individual measurements from pristine polyethylene (PE) pellets which were ground by Nanofract. X-rays were generated with a Rhodium (Rh) tube (M4 Tornado, Buker), explaining the oscillations between 2.5 and 3keV. Calcium (Ca) was clearly detected in all measurements. Inset (top right) shows an optical image of the PE pellets.

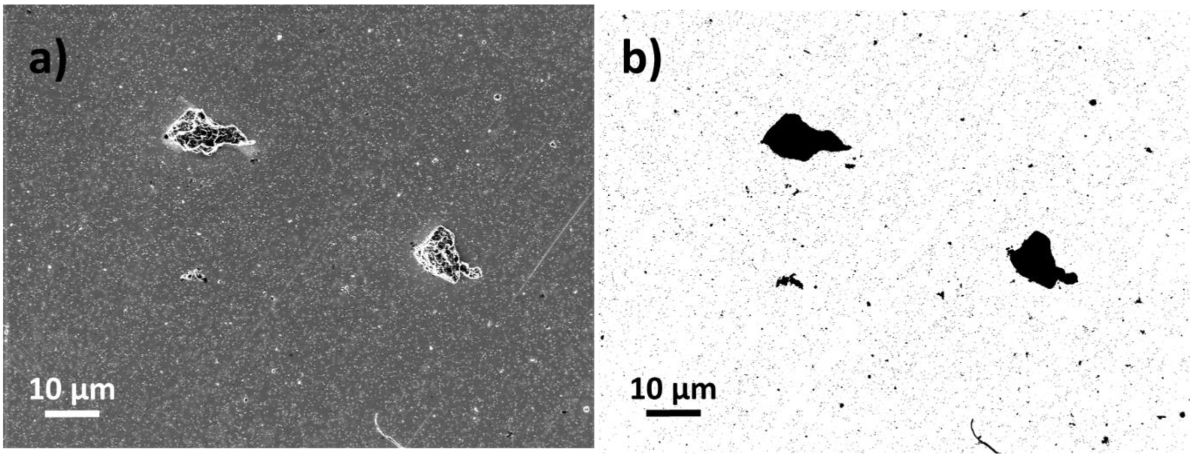

Fig. S3 Secondary electron (SE) image (InLens detector) (a) and backscattered electron (BSE) image (InLens energy selective backscattered electron (EsB)) detector (bias set to 1500V) (b) of a polyethylene (PE) – polyvinyl chloride (PVC) – soil extract deposited on a gold (40nm) and carbon (5nm) coated polycarbonate membrane. The SE image of the particles (a), reflecting their morphology / topography and thus consisting of a suite of different greyscales - is difficult to threshold. In the BSE image (b), reflecting the average atomic weight, the particles appear in an even greyscale (dark) and well separated from the bright background of the Au-coated polycarbonate membrane. The grey dots represent the holes of the polycarbonate membrane. Acceleration voltage: 3kV.

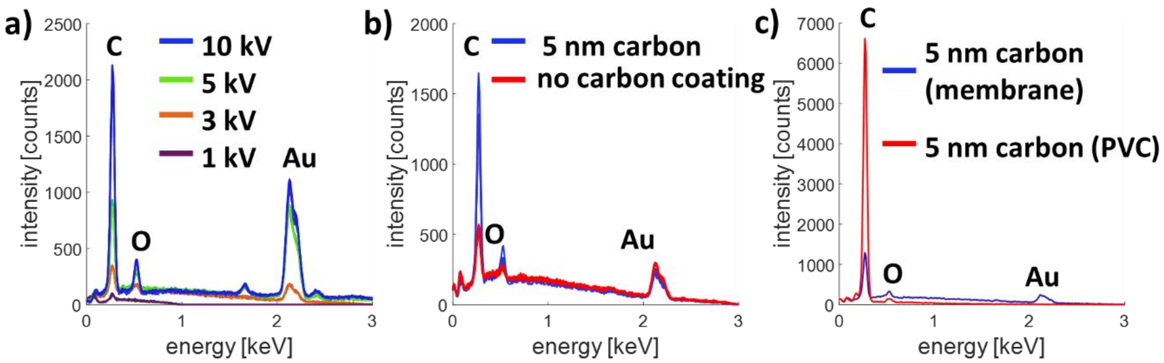

Fig. S4 a) Impact of increasing acceleration voltage (1, 3, 5, 10kV) on the x-ray signals of a polycarbonate (PC) membrane coated with 40nm of gold (Au). Spectra were recorded for 1s and using a current of 1nA and a process time of 4. b) Impact of an additional 5nm carbon (C) layer on top of the 40nm Au layer. c) Spectra from the PC membrane coated with 40nm Au and 5nm C (blue) and from a polyvinyl chloride (PVC) particle (diameter ~ 4µm) deposited on the Au coated PC membrane and coated with 5nm C. The spectra of b) and c) were recorded at an acceleration voltage of 3kV, a current of 800pA and a live time of 2s. The process time was set to '5'.

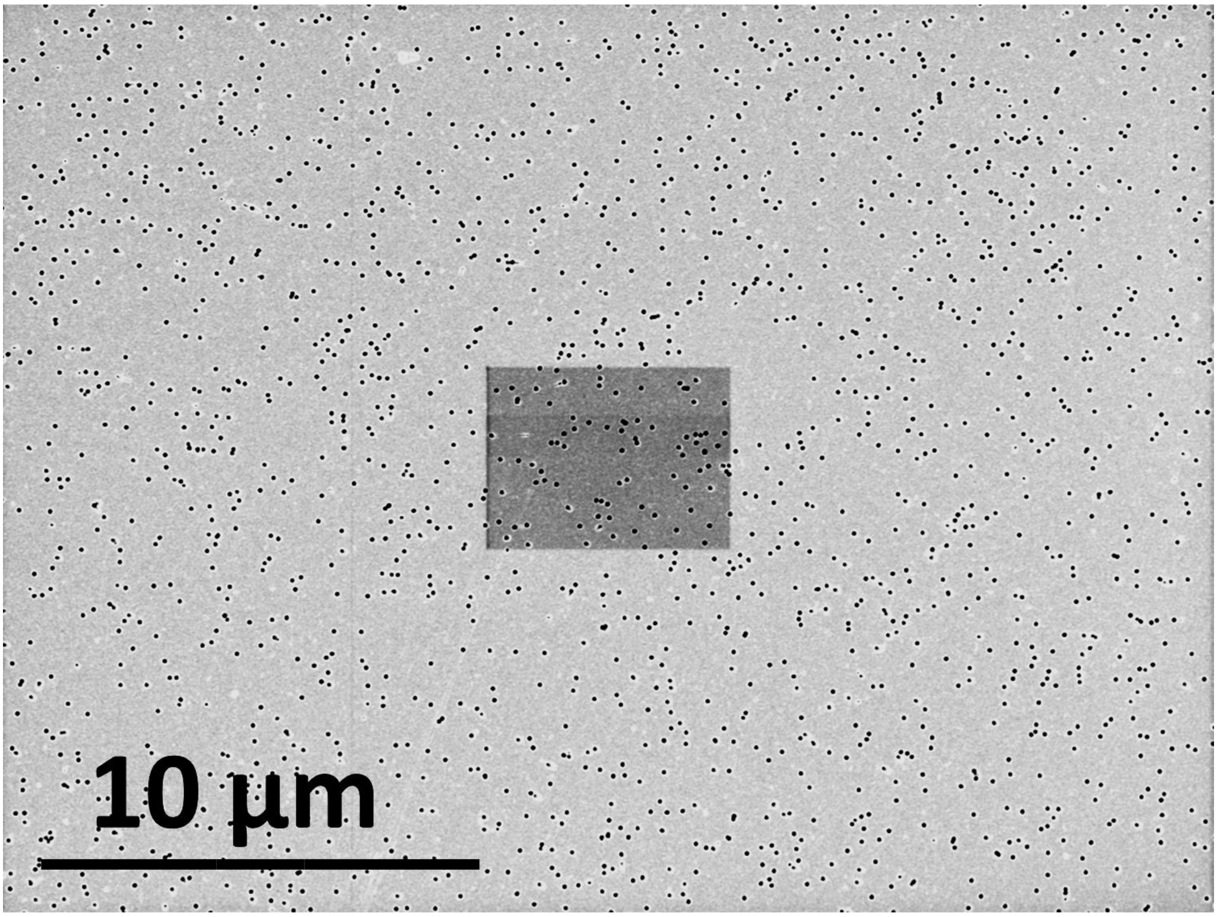

Fig. S5 Secondary electron image (InLense detector) of a gold (40nm) coated polycarbonate membrane, recorded after exposing a smaller area (dark area in the center of the image) to the electron beam. The dark area in the center of the image represents the carbon contamination that resulted from the previous exposure of this area to the electron beam (acceleration voltage: 3kV, current: 800pA).

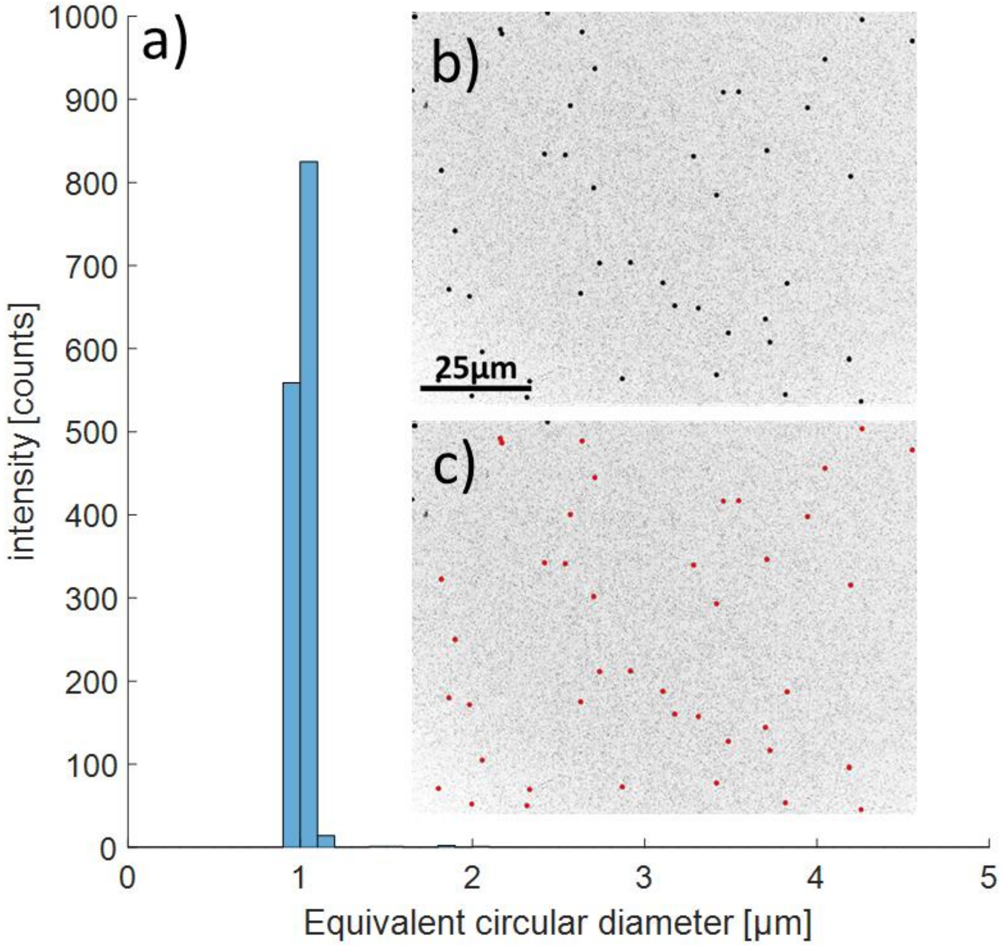

Fig. S6 a) Particle size distribution of 1µm polystyrene (PS) particles (Polybead® Microspheres (No: 07310-15), Polysciences, USA) from a stock suspension filtered on 40nm gold-coated polycarbonate membranes (0.1 µm pore diameter, 25mm diameter, Nucelopore). Total number of particles detected = 1868, mean diameter = 1.1µm (reported value from the manufacturer: 1.06µm). b) Backscattered electron image of the PS particles using the same operational conditions as for the main experiments and c) particles automatically detected (red dots) using the software package AZtec-Feature (AZtec v6.0, Oxford Instruments, UK).

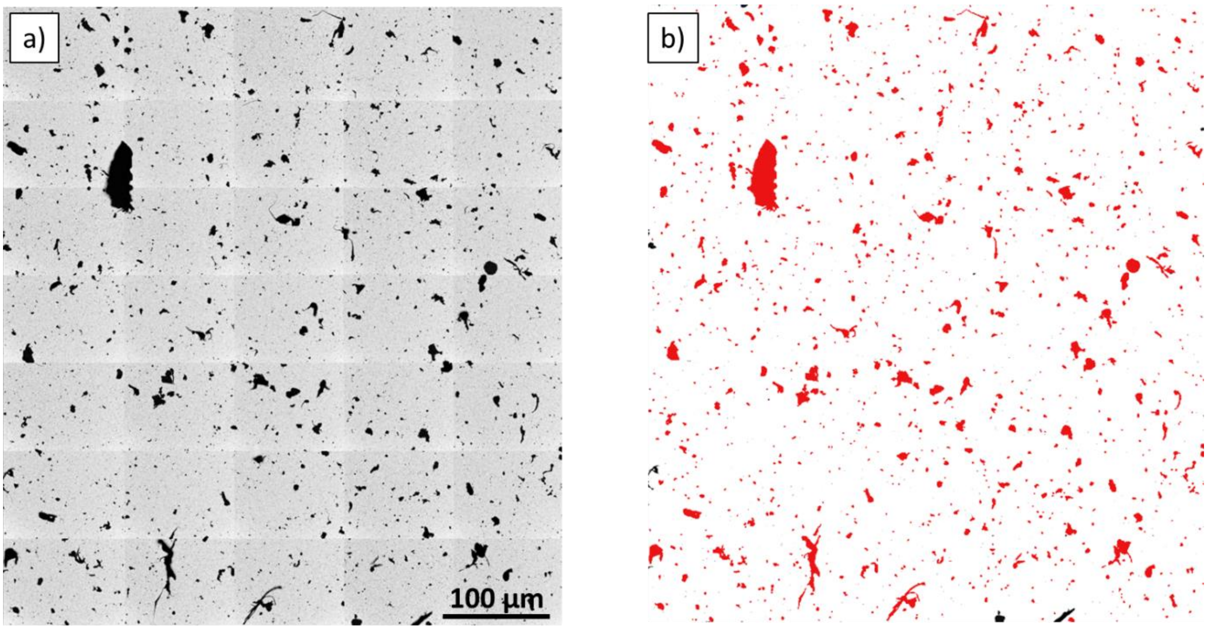

Fig. S7 a) Mosaic image of backscattered electron (BSE) images (7×5). Shown are particles from sample 7 (PE-PVC-SOIL 1:1:2, Table 1 in the main manuscript) deposited on a gold (40nm) coated polycarbonate membrane and coated with 5nm of carbon before analysis, b) identified particles are shown in red and the background has been removed for clarity.

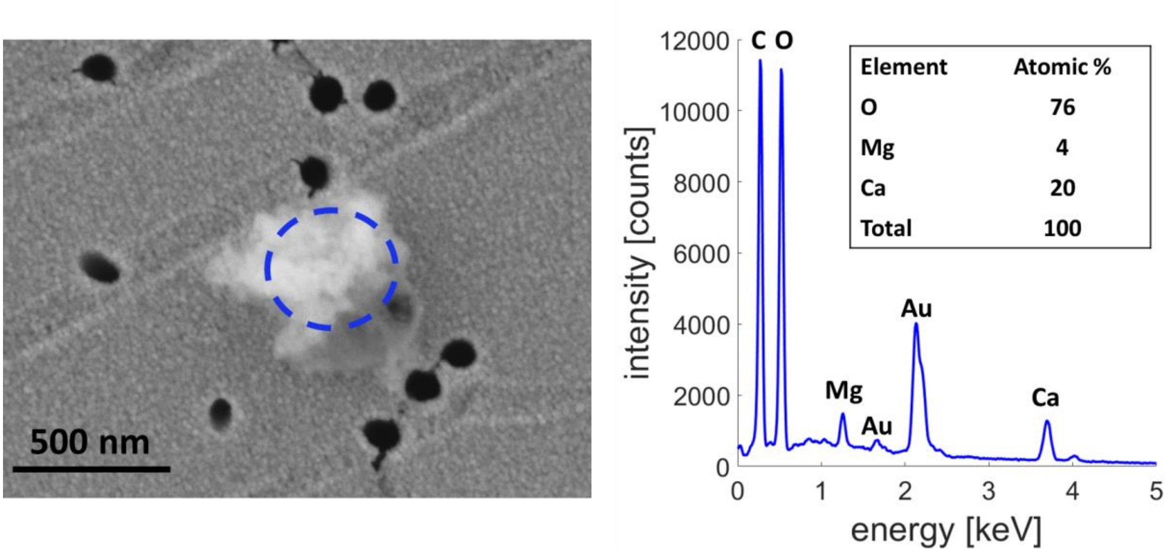

Fig. S8 Secondary electron image of a calcium carbonate particle (left) and energy dispersive x-ray spectrum (right) of the area marked with the dashed blue circle. The inset on the right shows the elemental composition of the particle, whereas carbon (C) and gold (Au) were used for deconvolution only. The stoichiometry of O:(Ca+Mg) = 3:1 fits well to the stoichiometry of (magnesium bearing)-calcite ((Ca,Mg)CO_3).

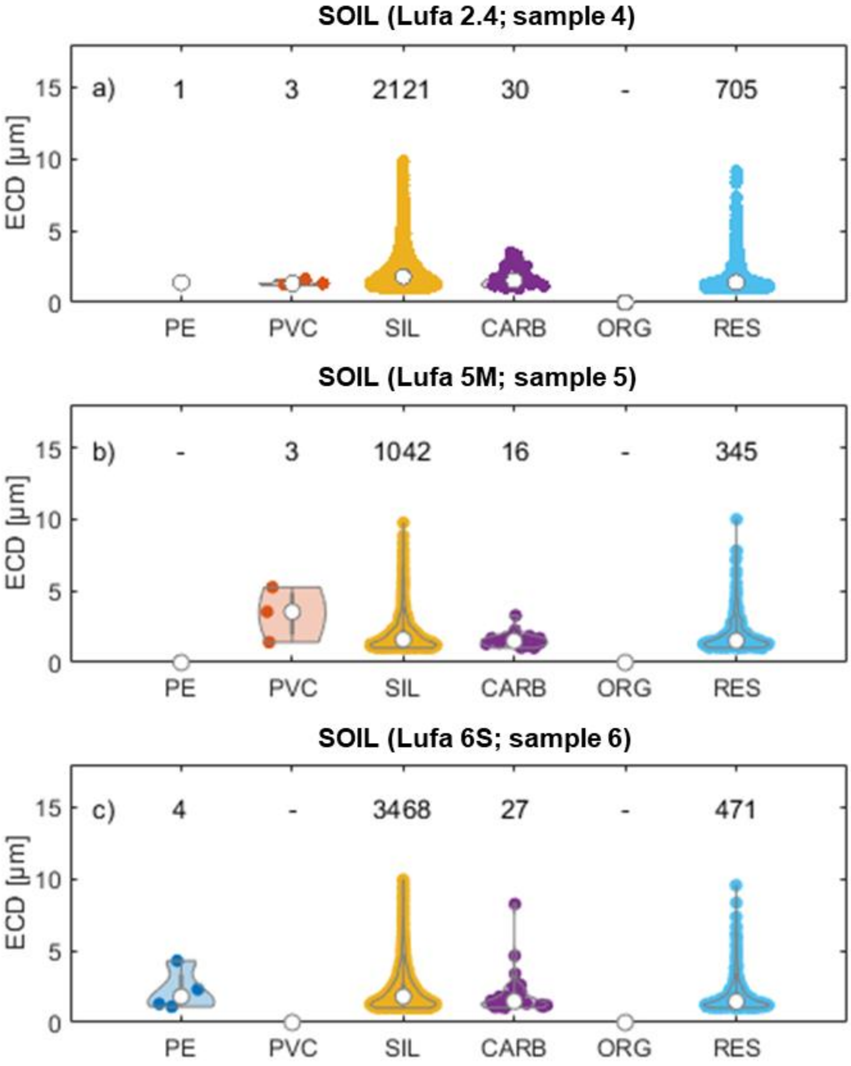

Fig. S9 Violin plots of particles detected in additional soil extracts a) LUFA 2.4 (sample 4), b) 5M (sample 5) and c) 6S (sample 6) filtered on gold coated (40nm) polycarbonate (PC) membranes. The PC membranes with the deposited particles were coated with an additional layer of carbon (5nm) before analysis. 'SIL' refers to silicate particles (which dominated the soil particles), 'CARB' to carbonates, 'ORG' to organic material containing nitrogen and 'RES' represents the residual particle category (as assigned in Fig. 2). Numbers on top of the violin plots refer to the number of particles of the corresponding category. A lower size threshold for the equivalent circular diameter (ECD) of 1µm and an upper threshold of 10µm was set for data analysis. Data with an upper size limit extended to 100µm are provided in Table S3 and Fig. S10B. See Table 1 for a description of the individual samples.

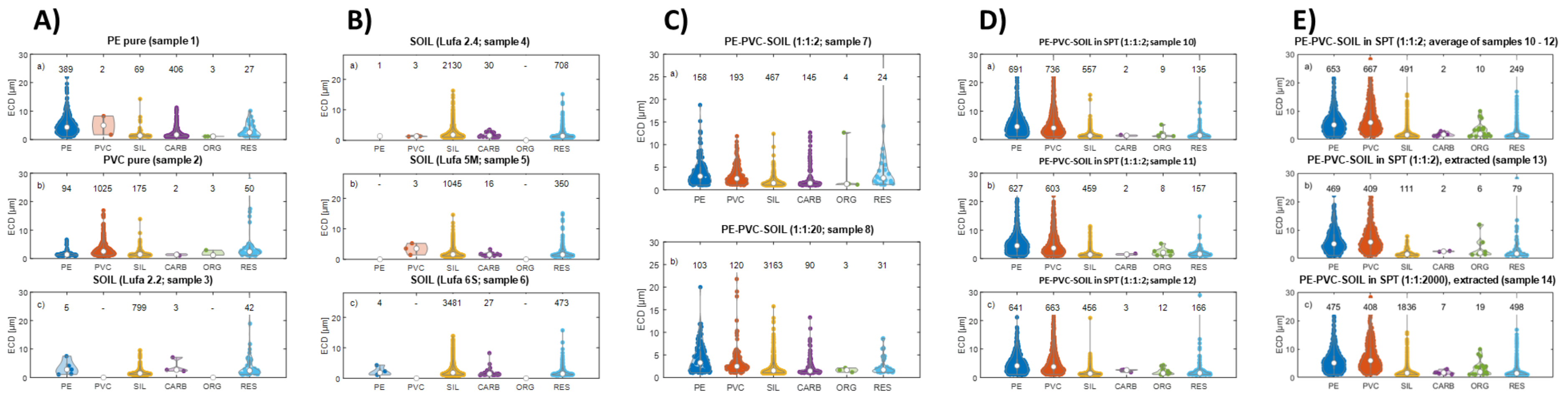

Fig. S10 Violin plots of the different particle categories including particle sizes between 1µm and 100µm. A): Stock suspensions of a) Polyethylene (PE, sample 1), b) Polyvinyl chloride (PVC, sample 2), c) Soil (LUFA 2.2, sample 3). B) Additional soils a) LUFA 2.4 (sample 4), b) LUFA 5M (sample 5) c) LUFA 6S (sample 6), C) Mixtures of PE, PVC and soil particles at ratios of a) 1 (PE) : 1 (PVC) : 2 (Soil) (sample 7) and b) 1 (PE) : 1 (PVC) : 20 (Soil) (sample 8). D) Triplicates (a-c) of a 1 (PE) : 1 (PVC) :2 (soil) mixture in sodium polytungstate (SPT) (samples 10-12). E) a) Average of triplicate measurements (from D), b) after density separation (sample 13) and c) after 1000× dilution in a soil suspension and following density separation (sample 14). Numbers on top of the individual violin plots refer to the number of detected particles in the respective categories. See Table 1 for a description of the individual samples. Volumes of PE and PVC stock suspension in D) and E) were always the same and thus, respective numbers directly represent the recoveries. 'SIL': silicate particles (which dominated the soil particles), 'CARB': carbonates, 'ORG': organic material containing nitrogen and 'RES': residual particle category (as assigned in Fig. 2). ECD: Equivalent circular diameter.

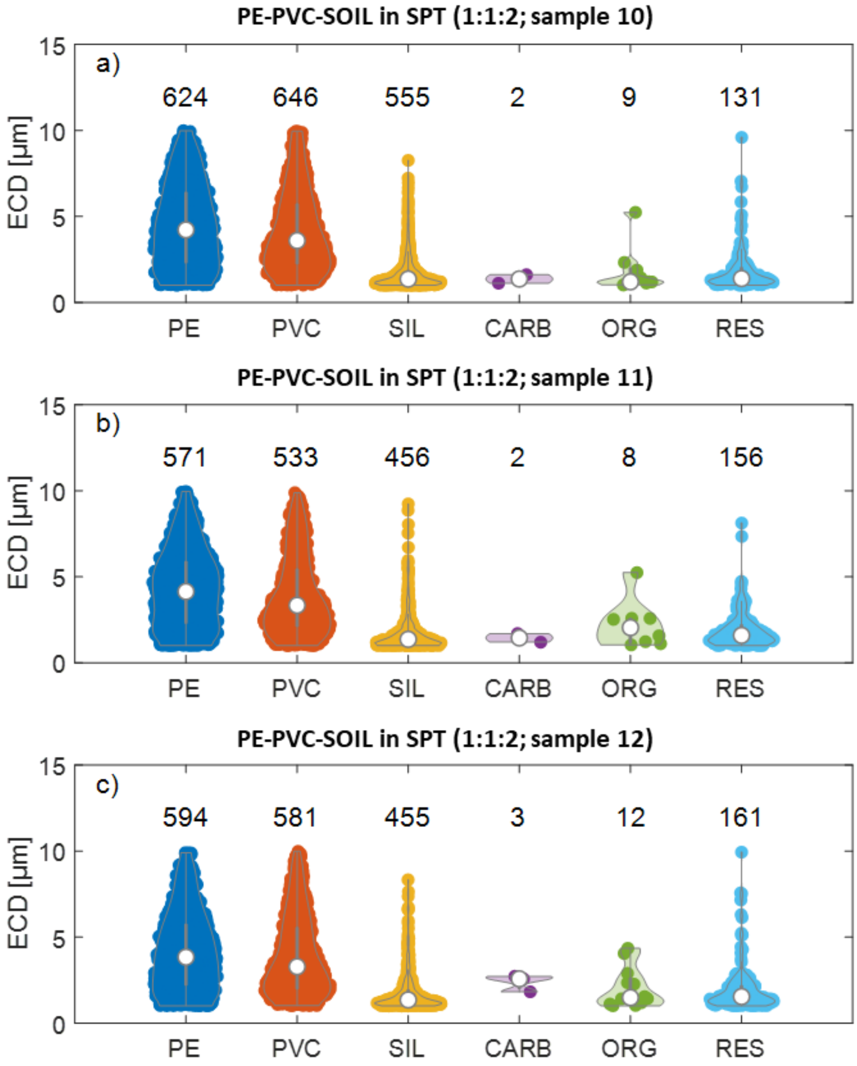

Fig. S11 Violin plots of the particles detected from mixtures of polyethylene (PE), polyvinyl chloride (PVC) and soil stock suspensions (nominal ratios: 1:1:2, all in sodium polytungstate) directly filtered on gold coated polycarbonate (PC) membranes. The PC membranes with the deposited particles were coated with a 5nm carbon layer before analysis. The three panels represent triplicate samples made from the same stock suspensions (samples 10-12). 'SIL' refers to silicate particles (which dominated the soil particles), 'CARB' to carbonates, 'ORG' to organic material containing nitrogen and 'RES' represents the residual particle category (as assigned in Fig. 2). Numbers on top of the violin plots refer to the number of particles of the corresponding category. A lower size threshold for the equivalent circular diameter (ECD) of 1µm and an upper threshold of 10µm was set for data analysis. Data with an upper size limit extended to 100µm are provided in the supporting information (Table S3 and Fig. S10D).

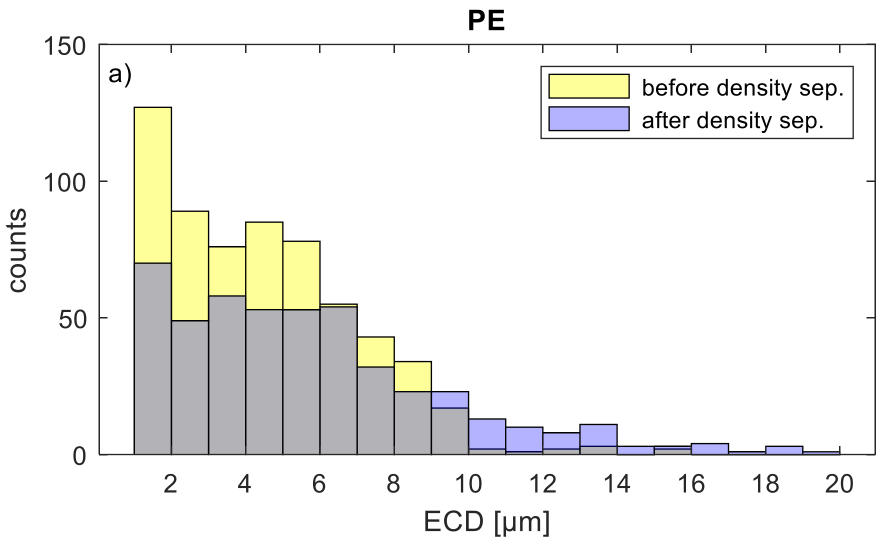

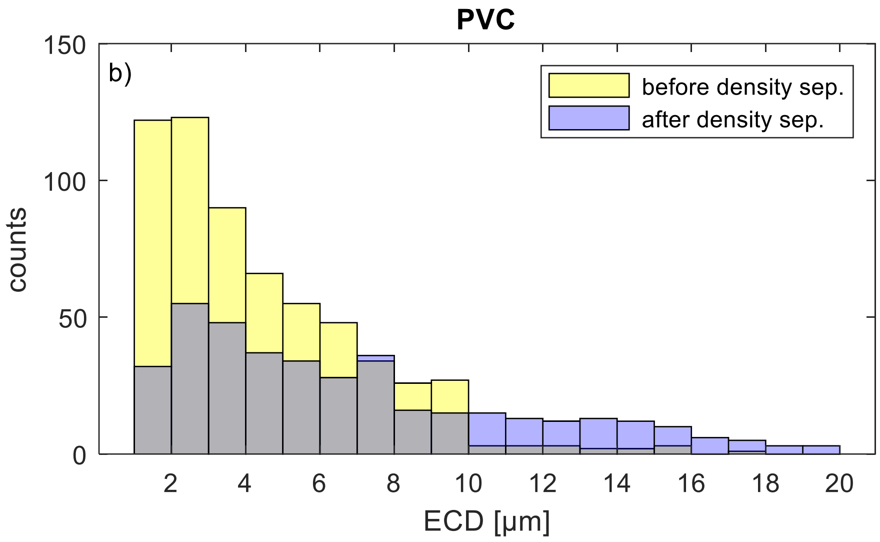

Fig. S12 Histogram of the equivalent circular diameters (ECD) of a) polyethylene (PE) and b) polyvinyl chloride (PVC) before density separation (samples 10-12, Table 1) and after the density separation (sample 13, Table 1).

Tables:

Table S1 Assignment of the major peaks of the polyethylene and the polyvinylchloride infrared absorption spectra, taken from [1, 2].

**Polyethylene**	
Absorption peak (cm^{-1})	Assignment
2918, 2850	CH_2 asymmetric stretch
1471	C-H bending
717	CH_2 rocking
**Polyvinylchloride**	
2970	stretching C-H of CHCl
2916	stretching C-H of CH_2
1427	deformation CH_2
1328, 1250	deformation C-H of CHCl
1095	stretching C-C
966	rocking CH_2
692	stretching C-Cl

Table S2: Experimental parameters and results from recovery experiments using polystyrene beads (Count Check Beads, No: 05-4011_R, Sysmex Partec GmbH). 'N detected' refers to the number of particles detected based on automated scanning electron microscopy analysis, 'conc. measured' refers to particle concentration calculated based on the number of particles detected and 'conc. stock' refers to the certified particle concentration of the stock suspension. The recovery was calculated relative to the stock suspension.

Sample	# sites [-]	# images/site [-]	#images [-]	image width [µm]	image height [µm]	area measured [mm^2]	filter diameter [mm]	filter area [mm^2]	filtered volume [mL]	N detected [#]	conc. meas [#/mL]	conc. stock [#/mL]	recovery [%]
CCB 01[a]	5	35	175	236	184	7.6	16	201.1	4.5	3431	20173	23140	87
CCB 02[a]	5	35	175	236	184	7.6	16	201.1	4.5	3478	20449	23140	88

[a]: measurements of duplicate filters

Table S3 Imaging parameters for the individual samples. In addition, the number of different particle types and the total number of particles detected are provided. N (X) refers to the number of particles detected in the respective particle class X, whereas PE: Polyethylene, PVC: Polyvinyl chloride, SIL: silicates, CARB: carbonates, ORG: organic particles, RES: residual particle category. The numbers in parentheses refer to particle numbers between 1µm and 100µm, the numbers in front of the parentheses refer to the particle number between 1µm and 10µm. Each site consists of 5×7 images, and each image was 113µm×88.5µm. Images were recorded with a 10% overlap. The corrected area/site, thus, comes to 0.29mm^2. IPA: isopropanol, EtOH: ethanol, SPT: sodium polytungstate.

sample number	description	# sites [-]	total area [mm^2]	N (PE) [#]	N (PVC) [#]	N (SIL) [#]	N (CARB) [#]	N (ORG) [#]	N (RES) [#]	N (Total) [#]
1[a]	PE pure	3	0.88	358 (389)	2 (2)	68 (69)	401 (406)	3 (3)	26 (27)	858 (896)
2[a]	PVC pure	5	1.47	94 (94)	1006 (1025)	174 (175)	2 (2)	3 (3)	43 (50)	1322 (1349)
3[a]	SOIL (Lufa 2.2)	3	0.88	5 (5)	0 (0)	799 (799)	3 (3)	0 (0)	38 (42)	845 (849)
4[a]	SOIL (Lufa 2.4)	5	1.47	1(1)	3(3)	2121(2130)	30(30)	0(0)	705(708)	2860(2872)
5[a]	SOIL (Lufa 5M)	5	1.47	0(0)	3(3)	1042(1045)	16(16)	0(0)	345(350)	1406(1414)
6[a]	SOIL (Lufa 6S)	5	1.47	4(4)	0(0)	3468(3481)	27(27)	0(')	471(473)	3970(3985)
7[a]	PE-PVC-SOIL (1:1:2)	1	0.29	148 (158)	191 (193)	466 (467)	141 (145)	3 (4)	22 (24)	971 (991)
8[a]	PE-PVC-SOIL (1:1:20)	1	0.29	97 (103)	113 (120)	3157 (3163)	88 (90)	3 (3)	31 (31)	3489 (3510)
9[b]	PE-PVC-SAND (2:1:3)[d]	5	1.47	440(446)	541(589)	1801(1805)	3(3)	34(34)	182(182)	3001(3059)
10[c]	PE-PVC-SOIL in SPT (1:1:2)[e]	5	1.47	624 (691)	646 (736)	555 (557)	2 (2)	9 (9)	131 (135)	1967 (2130)
11[c]	PE-PVC-SOIL in SPT (1:1:2)[e]	5	1.47	571 (627)	533 (603)	456 (459)	2 (2)	8 (8)	156 (157)	1726 (1856)
12[c]	PE-PVC-SOIL in SPT(1:1:2)[e]	5	1.47	594 (641)	581 (663)	455 (456)	3 (3)	12 (12)	161 (166)	1806 (1941)
13[c]	PE-PVC-SOIL in SPT (1:1:2), extracted	5	1.47	396 (469)	313 (409)	111 (111)	2 (2)	5 (6)	72 (79)	899 (1076)
14[c]	PE-PVC-SOIL in SPT (1:1:2000), extracted	5	1.47	415 (475)	301 (408)	1824 (1836)	7 (7)	19 (19)	479 (498)	3045 (3243)

[a]: Stock series 1: Individual suspensions of PE (~3,000#/µL), PVC (~9,000 #/µL) and soil particles (~60,000 #/µL), PE and soil prepared in EtOH, PVC prepared in IPA.

[b]: Stock series 2: Individual suspensions of PE (~500#/µL), PVC (~1,400 #/µL) and sand particles (~14 #/µL), PE and sand prepared in EtOH, PVC prepared in isopropanol IPA. Stocks made with sieved sand instead of soils and used for comparison with Raman measurements.

[c]: Stock series 3: Individual suspensions of PE (~800#/µL), PVC (~1,000 #/µL) and soil particles (~200,000 #/µL), all prepared in SPT solution (density 1.6g/mL).

[d]: Due to a shortage of PVC stock supension, a PE:PVC ratio of 1:1 was not achievable anymore.

[e]: Samples made in triplicates.

Table S4: Measured and calculated particle number concentrations, recoveries, and particle ratios for all samples for the size range 1-10µm. 'PE', 'PVC' and 'SIL' refers to polyethylene, polyvinyl chloride and silicates respectively. Particle concentrations of the stock suspensions ('c (PE, PVC, SIL)') in samples 1-3) were calculated based on the number of PE, PVC and SIL particles detected by automated scanning electron microscopy analyses ('[#]') in combination with the filtered volumes ('V') and number of areas analyzed ('N (areas)') (see eq(1) in section S2). Each area consisted of 7 x 5 images (each 113µm x 88.5µm) with an overlap of 10% (total area given in Table S3). The concentrations of the stock suspensions were then used to calculate the particle number concentrations ('c (PE, PVC, SIL)', the recoveries ('r (PE, PVC, SIL)', the expected particle numbers per filter ('N_e(PE, PVC, SIL, flt'), and the expected ratios ('$ratio_e$') in samples 7 and 8. The same approach was used for samples 10 – 14, whereas the average of the triplicate measurements (sample 10 – 12) was used to calculate the particle number concentrations of the stock suspension. 'N_m (PE, PVC, SIL, flt)' refers to the number of the respective particles on the filter derived from the measured number of particles of the respective filters. '[-]' refers to data which were either meaningless or not accessible.

sample number	description	PE [#]	V(PE) [µL]	PVC [#]	V(PVC) [µL]	SIL [#]	V(SIL) [µL]	N (areas) [#]	c (PE) [#/mL]	c (PVC) [#/mL]	c (SIL) [#/mL]	r(PE) [%]	r(PVC) [%]	r(SIL) [%]	N_m (PE,flt) [#]	N_e (PE,flt) [#]	N_m (PVC, flt) [#]	N_e (PVC,flt) [#]	N_m (SIL, flt) [#]	N_e (SIL,flt) [#]	$ratio_m$ (PE/PVC)	$ratio_e$ (PE/PVC)	$ratio_m$ (PE/SIL)	$ratio_e$ (PE/SIL)	$ratio_m$ (PVC/SIL)	$ratio_e$ (PVC/SIL)
1[a]	PE pure	358	200	2	0	68	0	3	4.07E+05	-	-	100	-	-	8.15E+04	8.15E+04	-	-	-	-	-	-	-	-	-	-
2[a]	PVC pure	94	0	1006	50	174	0	5	-	2.75E+06	-	-	100	-	-	-	1.37E+05	1.37E+05	-	-	-	-	-	-	-	-
3[a]	SOIL (Lufa 2.2)	5	0	0	0	799	10	3	-	-	1.82E+07	-	-	100	-	-	-	-	1.82E+05	1.82E+05	-	-	-	-	-	-
4[a]	SOIL (Lufa 2.4)	1	0	3	0	2121	10	5	-	-	2.90E+07	-	-	-	-	-	-	-	2.90E+05	-	-	-	-	-	-	-
5[a]	SOIL (Lufa 5M)	0	0	3	0	1042	10	5	-	-	1.42E+07	-	-	-	-	-	-	-	1.42E+05	-	-	-	-	-	-	-
6[a]	SOIL (Lufa 6S)	4	0	0	0	3468	10	5	-	-	4.74E+07	-	-	-	-	-	-	-	4.74E+05	-	-	-	-	-	-	-
7[a]	PE-PVC-SOIL (1:1:2)	148	200	191	50	466	20	1	5.05E+05	2.61E+06	1.59E+07	124	95	87	1.01E+05	8.15E+04	1.30E+05	1.37E+05	3.18E+05	3.64E+05	0.77	0.59	0.32	0.22	0.41	0.38
8[a]	PE-PVC-SOIL (1:1:20)	97	200	113	50	3157	200	1	3.31E+05	1.54E+06	1.08E+07	81	56	59	6.62E+04	8.15E+04	7.72E+04	1.37E+05	2.16E+06	3.64E+06	0.86	0.59	0.03	0.02	0.04	0.04
9[b]	PE-PVC-SAND (2:1:3)[d]	440	750	541	150	1801	20000	5	8.01E+04	4.93E+05	1.23E+04	-	-	-	6.01E+04	-	7.39E+04	-	2.46E+05	-	0.81	-	0.24	-	0.3	-
10[c]	PE-PVC-SOIL in SPT (1:1:2)[e]	624	300	646	250	555	2.6	5	2.84E+05	3.53E+05	2.92E+07	105	110	114	8.52E+04	8.14E+04	8.82E+04	8.01E+04	7.58E+04	6.67E+04	0.97	1.02	1.12	1.22	1.16	1.20
11[c]	PE-PVC-SOIL in SPT (1:1:2)[e]	571	300	533	250	456	2.6	5	2.60E+05	2.91E+05	2.40E+07	96	91	93	7.80E+04	8.14E+04	7.28E+04	8.01E+04	6.23E+04	6.67E+04	1.07	1.02	1.25	1.22	1.17	1.20
12[c]	PE-PVC-SOIL in SPT(1:1:2)[e]	594	300	581	250	455	2.6	5	2.70E+05	3.17E+05	2.39E+07	100	99	93	8.11E+04	8.14E+04	7.94E+04	8.01E+04	6.21E+04	6.67E+04	1.02	1.02	1.31	1.22	1.28	1.20
13[c]	PE-PVC-SOIL in SPT (1:1:2), extracted	396	300	313	250	111	2.6	5	1.80E+05	1.71E+05	5.83E+06	66	53	23	5.41E+04	8.14E+04	4.28E+04	8.01E+04	1.52E+04	6.67E+04	1.27	1.02	3.57	1.22	2.82	1.20
14[c]	PE-PVC-SOIL in SPT (1:1:2000), extracted	415	300	301	250	1824	2600	5	1.89E+05	1.64E+05	9.58E+04	70	51	0.4	5.67E+04	8.14E+04	4.11E+04	8.01E+04	2.49E+05	6.67E+07	1.38	1.02	0.23	0.0012	0.17	0.0012

[a]: Stock series 1: Individual suspensions of PE (~3,000#/µL), PVC (~9,000 #/µL) and soil particles (~60,000 #/µL), PE and soil prepared in EtOH, PVC prepared in IPA.

[b]: Stock series 2: Individual suspensions of PE (~500#/µL), PVC (~1,400 #/µL) and sand particles (~14 #/µL), PE and sand prepared in EtOH, PVC prepared in isopropanol IPA. Stocks made with sieved sand instead of soils and used for comparison with Raman measurements.

[c]: Stock series 3: Individual suspensions of PE (~800#/µL), PVC (~1,000 #/µL) and soil particles (~200,000 #/µL), all prepared in SPT solution (density 1.6g/mL).

[d]: Due to a shortage of PVC stock supension, a PE:PVC ratio of 1:1 was not achievable anymore.

[e]: Samples made in triplicates.

Table S5: Measured and calculated particle number concentrations, recoveries, and particle ratios for all samples for the size range 1-100µm. 'PE', 'PVC' and 'SIL' refers to polyethylene, polyvinyl chloride and silicates respectively. Particle concentrations of the stock suspensions ('c (PE, PVC, SIL)') in samples 1-3) were calculated based on the number of PE, PVC and SIL particles detected by automated scanning electron microscopy analyses ('[#]') in combination with the filtered volumes ('V') and number of areas analyzed ('N (areas)') (see eq(1) in section S2). Each area consisted of 7 x 5 images (each 113µm x 88.5µm) with an overlap of 10% (total area given in Table S3). The concentrations of the stock suspensions were then used to calculate the particle number concentrations ('c (PE, PVC, SIL)', the recoveries ('r (PE, PVC, SIL)', the expected particle numbers per filter ('N_e(PE, PVC, SIL, flt'), and the expected ratios ('$ratio_e$') in samples 7 and 8. The same approach was used for samples 10 – 14, whereas the average of the triplicate measurements (sample 10 – 12) was used to calculate the particle number concentrations of the stock suspension. 'N_m (PE, PVC, SIL, flt)' refers to the number of the respective particles on the filter derived from the measured number of particles of the respective filters. '[-]' refers to data which were either meaningless or not accessible.

sample number	description	PE [#]	V(PE) [µL]	PVC [#]	V(PVC) [µL]	SIL [#]	V(SIL) [µL]	N (areas) [#]	c (PE) [#/mL]	c (PVC) [#/mL]	c (SIL) [#/mL]	r(PE) [%]	r(PVC) [%]	r(SIL) [%]	N_m (PE,flt) [#]	N_e (PE,flt) [#]	N_m (PVC, flt) [#]	N_e (PVC,flt) [#]	N_m (SIL, flt) [#]	N_e (SIL,flt) [#]	$ratio_m$ (PE/PVC)	$ratio_e$ (PE/PVC)	$ratio_m$ (PE/SIL)	$ratio_e$ (PE/SIL)	$ratio_m$ (PVC/SIL)	$ratio_e$ (PVC/SIL)
1[a]	PE pure	389	200	2	0	69	0	3	4.43E+05	-	-	100	-	-	8.86E+04	8.86E+04	-	-	-	-	-	-	-	-	-	-
2[a]	PVC pure	94	0	1025	50	175	0	5	-	2.80E+06	-	-	100	-	-	-	1.40E+05	1.40E+05	-	-	-	-	-	-	-	-
3[a]	SOIL (Lufa 2.2)	5	0	0	0	799	10	3	-	-	1.82E+07	-	-	100	-	-	-	-	1.82E+05	1.82E+05	-	-	-	-	-	-
4[a]	SOIL (Lufa 2.4)	1	0	3	0	2130	10	5	-	-	2.91E+07	-	-	160	-	-	-	-	2.91E+05	-	-	-	-	-	-	-
5[a]	SOIL (Lufa 5M)	0	0	3	0	1045	10	5	-	-	1.43E+07	-	-	78	-	-	-	-	1.43E+05	-	-	-	-	-	-	-
6[a]	SOIL (Lufa 6S)	4	0	0	0	3481	10	5	-	-	4.75E+07	-	-	261	-	-	-	-	4.75E+05	-	-	-	-	-	-	-
7[a]	PE-PVC-SOIL (1:1:2)	158	200	193	50	467	20	1	5.40E+05	2.64E+06	1.59E+07	122	94	88	1.08E+05	8.86E+04	1.32E+05	1.40E+05	3.19E+05	3.64E+05	0.82	0.63	0.34	0.24	0.41	0.38
8[a]	PE-PVC-SOIL (1:1:20)	103	200	120	50	3163	200	1	3.52E+05	1.64E+06	1.08E+07	79	59	59	7.03E+04	8.86E+04	8.20E+04	1.40E+05	2.16E+06	3.64E+06	0.86	0.63	0.03	0.02	0.04	0.04
9[b]	PE-PVC-SAND (2:1:3)[d]	446	750	589	150	1805	20000	5	8.12E+04	5.36E+05	1.23E+04	-	-	-	6.09E+04	-	8.04E+04	-	2.47E+05	-	0.76	-	0.25	-	0.33	-
10[c]	PE-PVC-SOIL in SPT (1:1:2)[e]	691	300	736	250	557	2.6	5	3.15E+05	4.02E+05	2.93E+07	106	110	114	9.44E+04	8.92E+04	1.01E+05	9.11E+04	7.61E+04	6.70E+04	0.94	0.98	1.24	1.33	1.32	1.36
11[c]	PE-PVC-SOIL in SPT (1:1:2)[e]	627	300	603	250	459	2.6	5	2.85E+05	3.29E+05	2.41E+07	96	90	94	8.56E+04	8.92E+04	8.24E+04	9.11E+04	6.27E+04	6.70E+04	1.04	0.98	1.37	1.33	1.31	1.36
12[c]	PE-PVC-SOIL in SPT(1:1:2)[e]	641	300	663	250	456	2.6	5	2.92E+05	3.62E+05	2.40E+07	98	99	93	8.76E+04	8.92E+04	9.06E+04	9.11E+04	6.23E+04	6.70E+04	0.97	0.98	1.41	1.33	1.45	1.36
13[c]	PE-PVC-SOIL in SPT (1:1:2), extracted	469	300	409	250	111	2.6	5	2.14E+05	2.23E+05	5.83E+06	72	61	23	6.41E+04	8.92E+04	5.59E+04	9.11E+04	1.52E+04	6.70E+04	1.15	0.98	4.23	1.33	3.68	1.36
14[c]	PE-PVC-SOIL in SPT (1:1:2000), extracted	475	300	408	250	1836	2600	5	2.16E+05	2.23E+05	9.64E+04	73	61	0.4	6.49E+04	8.92E+04	5.57E+04	9.11E+04	2.51E+05	6.70E+07	1.16	0.98	0.26	0.00	0.22	0.00

[a]: Stock series 1: Individual suspensions of PE (~3,000#/µL), PVC (~9,000 #/µL) and soil particles (~60,000 #/µL), PE and soil prepared in EtOH, PVC prepared in IPA.

[b]: Stock series 2: Individual suspensions of PE (~500#/µL), PVC (~1,400 #/µL) and sand particles (~14 #/µL), PE and sand prepared in EtOH, PVC prepared in isopropanol IPA. Stocks made with sieved sand instead of soils and used for comparison with Raman measurements.

[c]: Stock series 3: Individual suspensions of PE (~800#/µL), PVC (~1,000 #/µL) and soil particles (~200,000 #/µL), all prepared in SPT solution (density 1.6g/mL).

[d]: Due to a shortage of PVC stock supension, a PE:PVC ratio of 1:1 was not achievable anymore.

[e]: Samples made in triplicates.

Table S6 Comparison of results obtained from automated scanning electron microscopy energy dispersive x-ray analysis (SEM-EDX) and automated Raman microspectroscopy measurements. PE: Polyethylene, PVC: Polyvinyl chloride. 'N_m (PE,PVC, SAND)' refers to the number of particles detected within a certain area ('A_m'). 'V (PE, PVC, SAND)' refers to the volume of respective stock suspensions that were used to prepare the samples. 'N_A (PE, PVC)' refers to the number of particles per mm^2 and 'recovery (PE, PVC)' refers to the abundance of PE and PVC particles detected by automated Raman analysis relative to the amount detected by automated SEM-EDX analysis.

method	N_m (PE) [#]	V (PE) [µL]	N_m (PVC) [#]	V (PVC) [µL]	N_m (SAND) [#]	V (SAND) [µL]	A_m [mm^2]	N (PE) [#/mm^2]	N (PVC) [#/mm^2]	recovery (PE) [%]	recovery (PVC) [%]
SEM-EDX	446	750	589	150	1805	20000	1.47	303	400	-	-
RAMAN[a]	434	750	228	150	NA	NA	1.44	302	159	**100**	**40**
RAMAN[a]	366	750	240	150	NA	NA	1.44	255	167	**84**	**42**
RAMAN[a]	428	750	247	150	NA	NA	1.44	298	172	**98**	**43**

[a]: triplicate measurements on the same filter

1. Beltrán M, Marcilla A (1997) FOURIER TRANSFORM INFRARED SPECTROSCOPY APPLIED TO THE STUDY OF PVC DECOMPOSITION. European Polymer Journal 33:1135–1142. https://doi.org/10.1016/S0014-3057(97)00001-3

2. Gulmine JV, Janissek PR, Heise HM, Akcelrud L (2002) Polyethylene characterization by FTIR. Polymer Testing 21:557–563. https://doi.org/10.1016/S0142-9418(01)00124-6
